# Incubation and grazing effects on spirotrich ciliate diversity inferred from molecular analyses of microcosm experiments

**DOI:** 10.1371/journal.pone.0215872

**Published:** 2019-05-06

**Authors:** Jean-David Grattepanche, Doris L. Juarez, Cameah C. Wood, George B. McManus, Laura A. Katz

**Affiliations:** 1 Department of Biological Sciences, Smith College, Northampton, Massachusetts, United States of America; 2 Department of Marine Sciences, University of Connecticut, Groton, Connecticut, United States of America; 3 Program in Organismic and Evolutionary Biology, University of Massachusetts, Amherst, Massachusetts, United States of America; Helmholtz-Zentrum fur Umweltforschung UFZ, GERMANY

## Abstract

We used an experimental approach of analyzing marine microcosms to evaluate the impact of both predation (top-down) and food resources (bottom-up) on spirotrich ciliate communities. To assess the diversity, we used two molecular methods–denaturing gradient gel electrophoresis (DGGE) and high-throughput sequencing (HTS). We carried out two types of experiments to measure top-down (adult copepods as predators) and bottom-up effects (phytoplankton as food resources) on the spirotrich ciliates. We observed both strong incubation effects (untreated controls departed from initial assessment of diversity) and high variability across replicates within treatments, particularly for the bottom-up experiments. This suggests a rapid community turn-over during incubation and differential susceptibility to the effects of experimental manipulation. Despite the variability, our analyses reveal some broad patterns such as (1) increasing adult copepod predator abundance had a greater impact on spirotrich ciliates than on other microbial eukaryotes; (2) there was no evidence for strong food selection by the dominant spirotrich ciliates.

## Introduction

The planktonic food web is the base of aquatic ecosystems, and hence strongly impacts the productivity and health of approximately two thirds of our planet. Diversity within the planktonic food web is great: prokaryotes recycling dissolved organic matter and small phytoplankton are eaten by heterotrophic microeukaryotes such as nanoflagellates and ciliates [1–3]. These predators and larger phytoplankton serve in their turn as prey for larger organisms, such as larger ciliates, dinoflagellates, and small metazoans including copepods, which are themselves consumed by larger invertebrates and fish [1, 2, 4]. Given these complex interactions, disentangling dynamics in planktonic food webs is difficult but essential for understanding both ecosystem function and health [5–7].

To date, investigations have focused on functional compartments that represent phytoplankton, mesozooplankton, and microzooplankton [8–15]; studies looking at species-level relationships, particularly within the microzooplankton, are sparse [16–19]. These interactions are difficult to observe in a quantitative way because of the inherent limitations of microscopy in terms of the number of samples that can be examined and the depth of sampling possible. Here, we use molecular methods to analyze the impact of manipulations of copepod and phytoplankton abundance on the diversity of micrograzers in the SAR (Stramenopila, Alveolata, Rhizaria) eukaryote clade, with a particular focus on spirotrich ciliates.

The bulk of eukaryotic diversity is microbial, with larger organisms (plants, animals and fungi) representing just three of about 75 lineages (e.g., [20, 21, 22]). Among the major clades of eukaryotes, SAR represents up to half of eukaryotic diversity [23, 24]. SAR includes diverse parasites (e.g. the oomycetes), algae (e.g. diatoms, dinoflagellates, kelp), heterotrophic predators (e.g., ciliates, cercozoa) and many less well known lineages [24]. While SAR members are both widespread and abundant, there is still much to investigate about this eukaryotic clade including a large “dark area”, which includes sequences from the environment that are not linked to documented morphology and morphospecies that have not yet been sequenced [24].

One focus of this study is the ciliates of the class Spirotrichea (SAR, Alveolata), which includes the most quantitatively important clades of planktonic grazers [16, 18, 25]. They can consume up to 100% of the standing stock of nanoplankton every day [3, 4, 16, 17, 26], but species-by-species interactions within these predator/prey assemblages remain largely unknown. Our study used copepods as a model predator for assessing top-down (TD) effects in the planktonic food web, in particular how this interaction affects diversity and composition of spirotrich ciliate assemblages. Previous studies have shown that copepod diets are composed up to 50% of ciliates [27–30], with bias towards energy-rich heterotrophic lineages [31].

Phytoplankton blooms, defined as a high abundance of one or a few phytoplankton species, have major impacts on the oceans and have been increasing globally in recent years (reviewed in [32–36]). Mechanisms behind the occurrence of blooms are varied, and may include decoupling of predator/prey dynamics [37], cascading interactions among grazers (e.g. ciliates grazed by copepods cannot regulate phytoplankton growth), parasitism [38, 39] or physical factors [40]. Here we used monospecific additions of nanophytoplankton as models for assessing bottom-up (BU) effects on ciliate grazers during simulated bloom events.

To assess the impact of copepod and phytoplankton abundance on all-eukaryote, SAR and ciliate assemblages, we carried out microcosm experiments and analyzed the community composition using both a fingerprinting technique (denaturing gradient gel electrophoresis; DGGE) for all eukaryotes and for ciliates in the class Spirotrichea, and high throughput sequencing (HTS) with primers designed for the whole SAR eukaryotic clade, which includes ciliates. We examined potential biases of our methods and then tested the hypotheses that (1) increased copepod predation changes the ciliate community composition by selective predation on specific lineages, and (2) phytoplankton abundance and composition have a predictable impact on the whole SAR assemblage, and on ciliates in particular.

## Material and methods

### Collection and setup

We conducted two types of microcosm experiments using natural plankton assemblages: one focusing on predation effects during the summer 2013 (three “top-down” experiments), and one on the impact of simulated phytoplankton blooms in the fall of 2014 (three “bottom-up” experiments; Table 1). Our sampling site was the dock at the University of Connecticut Avery Point, Groton CT (Long Island Sound; 41°18'59"N, 72°03'39"W). For each experiment, we sampled ~20L of seawater from the surface and prescreened through a 200μm mesh in order to remove the mesozooplankton, in particular adult copepods. At sampling, we measured seawater temperature and salinity (Table 1).

**Table 1:** Summary of microcosm experiments conditions and molecular methods used.

Microcosm	Start	Starting salinity	Starting T (ºC)	Duration (days)	Final salinity	Final T (ºC)	Copepods	Molecular methods
TD 1	10 Jul 13	26	23	2	26	23	*Acartia tonsa*	*DGGE spiro & alleuk*
TD 2	15 Jul 13	30	20	3	30	20	*Acartia tonsa*	*DGGE spiro & alleuk*
TD 3	3 Jul 13	23	23	6	32	19	*Acartia hudsonica*	*DGGE spiro & alleuk*
BU 1	10 Nov 14	33	12	3	32	14	adults removed	*DGGE spiro*
BU 2	11 Nov 14	32	13	3	32	13	adults removed	*DGGE spiro*
BU 3	14 Nov 14	32	12	3	32	12	adults removed	*DGGE spiro* & HTS

To estimate the ciliate diversity *in situ* (hereafter referred to as the starting community or T_0_), we filtered and preserved 500mL of prescreened seawater (<200µm). Samples were either collected directly on a 3µm pore size nitrocellulose filter (top-down experiment) or filtered in series through 80μm mesh (to avoid metazoan DNA), 10 and 2μm polycarbonate filters (bottom-up experiment). All of the filters were immediately placed into DNA prep buffer (100mM NaCl, Tris-EDTA at pH 8, and 0.5% of SDS) and stored at 4ºC.

We used dialysis tubing (cellulose membrane that is pervious to molecules <12,000 molecular weight; product D9402, Sigma) closed with plastic clips at both ends to contain c. 500 mL, as our microcosms. The glycerol used as humectant of the dialysis tubing was removed by soaking in multiple rinses of tap water overnight before the experiments. The microcosms were incubated in a sea table with continuous *in situ* seawater circulation for temperature control [41]. The use of dialysis tubing allows the exchange of oxygen, nutrients, and other metabolites between the inside of the bag and the *in situ* seawater of the sea table, but the organisms are not able to escape.

#### Top-down experiment (TD)

Three treatments were used during the top-down experiment to assess the impact of adult copepod grazing on spirotrich ciliates: no adult copepods, five adult copepods, and ten adult copepods (labelled thereafter as C for control, N for ‘natural’ predation pressure and H for High predation pressure, respectively) were added i.e. copepods abundance of 0, 10 and 20 ind.L^-1^, which is in the range of the natural abundance of copepods in this temperate system. The high copepod abundance of 20 ind.L^-1^ is at the high end of copepod abundance in Long Island Sound and the North Atlantic [16, 42–44]. For each treatment, dialysis tubes were filled with 500 mL of the starting community (*in situ* seawater <200µm). Each treatment was made in triplicate, and the experiment was performed three times. The copepods used for the experiment (*Acartia hudsonica [winter-spring species]*, or *Acartia tonsa [summer-fall species]* [44, 45] were picked from cultures maintained at the University of Connecticut Department of Marine Sciences, except for 10 July 2013, which used wild-caught *A*. *tonsa* from a plankton tow (Table 1).

#### Bottom-up experiment (BU)

For the bottom-up experiment, we simulated separate blooms using three phytoplankton cultures: the diatom *Phaeodactylum tricornutum*, the haptophyte *Isochrysis galbana*, and the chlorophyte *Tetraselmis chui*. These three algae were from the culture collection of the National Marine Fisheries Service Laboratory in Milford CT (USA). We chose these three phytoplankton species because spirotrich cultures can be grown on them. In order to assess the effect of high-levels of phytoplankton on spirotrich ciliate community composition, we incubated the starting community (*in situ* seawater < 200µm) without phytoplankton added (control; just the natural <200 µm phytoplankton assemblage), or with 10^4^ cells mL^-1^ of one of the three phytoplankton cultures added (i.e. the diatom, the haptophyte or the chlorophyte culture), for a total of four treatments. Each treatment was done in duplicate or triplicate, and the experiment was carried out three times during the fall of 2013. The microcosms were incubated under two layers of neutral density screen (216 µmol photons m^-2^s^-1^, or 0.27 of surface irradiance) in the sea table.

#### Incubation duration

We were not sure of the time scales for grazing interactions, especially if multiple trophic transfers (cascades) were involved, so the different top-down experiments were incubated for two, three, or six days while the bottom-up experiments were all incubated for three days (see Table 1). We estimated that the latter (three days) would be sufficient time (2–3 generations for spirotrich ciliates) to observe changes in microbial eukaryote assemblages. Regarding the top-down incubation, the generation time for *Acartia* spp. is 13-15d at 20˚C [46], so we expected that the longest incubation (six days) would not induce a strong increase of the number of adult copepods (developing from larval stages present in our < 200µm in situ seawater). After incubation, copepods were removed from the top-down experiments with 200 µm mesh and enumerated. For DNA, 300-400mL of each bag was filtered through 3μm nitrocellulose filter (top-down experiments) or 10 and 2μm polycarbonate membranes in series (bottom-up experiments; Table 1). The bottom-up experiments thus had additional information about diversity in different size-fractions.

### Molecular assessment of planktonic diversity

#### DNA extraction and amplification

The DNA extraction and amplification methods are detailed in Grattepanche et al [47] and Sisson et al [48]. In summary, DNA was extracted using a phenol chloroform protocol adapted for filters [49] for top-down experiments or using ZR Soil Microbe DNA MiniPrep kit following the instructions given by the manufacturer (Zymo Research, CA) for bottom-up experiments. Two sets of primers were used for each experiment to amplify (S1 Table): a 350bp SSU-rDNA fragment specific to spirotrich ciliates (528- with GC clamp and 152+; [50]) for both experiments as the main focus of this study. To observe if the overall microbial eukaryote community was impacted during our top-down experiments, a 300bp SSU-rDNA fragment of ‘all’ eukaryotes [51] was also used. For the same reason and to observe more rare species compared to DGGE, a 150bp SSU-rDNA fragment specific to the SAR clade was amplified and sequenced by HTS [48] for one of the bottom-up experiments (Table 1). The amplifications were performed with the Q5 enzyme and either a 1:10 or 1:100 dilution of total DNA. Up to 5 PCR products were pooled to avoid PCR biases (over or under-amplification of some sequences, chimeras, etc.; see [52] for discussion of these issues).

#### DGGE as a tool for assessment of abundant community members

Denaturing Gradient Gel Electrophoresis (DGGE) is a DNA fingerprinting method that detects variation in DNA sequence composition from PCR products separated on an acrylamide gel containing a denaturing gradient of 35 to 55% urea-deionized formamide [47]. We used DGGE to assess the composition of abundant species, as in past studies. DGGE gives good resolution for changes in spirotrich ciliate community composition [47, 53], and has shown similar patterns to those found by HTS [54]. We conducted DGGE using two sets of primers: Spirotrichea ciliate- specific primers ([50]; referred to as DGGE spiro) for all experiments, and eukaryote-specific primers ([51]; referred to as DGGE alleuk) only for the top-down experiments (Table 1). Gels were incubated for at least 15 hours under 45 volts (DCode Universal Mutation Detection System user guide; Bio-Rad). After incubation, they were stained using SYBR Gold and documented using a Kodak imager.

To assess the taxonomic community composition of the spirotrich ciliates, bright bands within the gels were excised, and DNA was eluted in 10μL of TE buffer overnight at 4°C. The resulting DNA was amplified for 10 cycles using the same set of primers without the GC clamp (S1 Table). PCR products were cleaned with ExoSap It (Thermo Fisher Scientific) and sequenced using the Big Dye Terminator v3.1 Cycle Sequencing Kit (Life technologies). Sanger sequencing was performed at the Smith College Center for Molecular Biology or at the University of Rhode Island Genomics and Sequencing Center. A total of 84 DGGE bands were sequenced for this study. The PCR and DGGE were carried out multiple times to ensure that our results were robust in estimating abundant community members. DGGEs with the pooled PCR products generated the same band pattern for each sample replicated, consistent with our previous DGGE studies [47, 53, 55].

#### High throughput sequencing and bioinformatics

Prior to the high-throughput sequencing (bottom-up experiment 3 only), PCR amplicons from the SAR primers were pooled and cleaned using Agencourt AMPure XP beads (Beckman Coulter Life Sciences). Sequencing was performed at the University of Rhode Island Genomics and Sequencing Center for Amplicon Sequencing. The amplicons were multiplexed and sequenced for 2x150 cycles using Illumina MiSeq.

HTS data analysis used the same four-step bioinformatic pipeline implemented and described in Sisson et al [48]. In summary, the first step assembled forward and reverse reads using Paired-End reAd mergeR (PEAR, [56]). During the paired-end assembling, ambiguous bases were removed, and reads were trimmed with an Illumina quality score of 33 (maximal score 40) and an overlapping cutoff of 120 bases (i.e. the overlap between the forward and reverse reads had to be at least 120 bp). During the second step, OTUs were built with the clustering algorithm SWARM (v 2.1.9, [57]) using the standard distance (d = 1, the default value). In order to remove sequencing errors, only OTUs with more than 5 reads were included in subsequent analyses. Chimeras were identified and discarded using UCHIME with default parameters [58]

Taxonomy was assigned to each of the remaining OTUs using a BLAST approach *via* custom script with cutoff at 95% identity similarity, 80% of coverage, and 2e^-50^ E-value. OTUs were aligned with our curated SAR alignment in MAFFT using the '—add fragment' algorithm [59]. Columns with more than 75% missing data (e.g. insertion present in only one of four species) were removed to save computing time during tree building, then a RAxML tree was built using GTR-GAMMA-I parameters on CIPRES [60] to enable outgroup removal (non-SAR OTUs). The outgroups were checked by eye and discarded. After this round of cleaning, the remaining reads were subsampled at 60,000 reads to enable us to compare samples. A second taxonomic assignment was performed using a phylogenetic approach and OTUs were assigned to the sister closest on the tree (step 4). We performed a dual taxonomic assignment to avoid mis-assignment, and more importantly to be able to add a taxonomy to each OTU even if there is no close relative by BLAST.

### Statistical analyses

DGGE gels were analyzed by eye and only presence/absence data are considered. Species richness (number of abundant species) was compared using Pearson’s correlation coefficient and t-tests. Similarity of biological replicates and relationship between Spirotrichea richness and environment (number of eukaryotes, number of copepods, number of SAR species) were analyzed with regression analysis (ANOVA). Band patterns (abundant members of the community) and replicability were compared by non-metric multidimensional scaling (NMDS) on the Jaccard binary index (presence/absence diversity index) using the phyloseq and vegan packages [61, 62] implemented in R [63]. Patterns within the HTS data were analyzed using the Unifrac dissimilarity index, which considers the phylogenetic distance between taxa [64], or the Bray-Curtis dissimilarity index. Principal coordinate analysis (PCoA), Permutational multivariate analysis of variance (PERMANOVA), and pairwise comparison with Bonferroni correction were performed in R [63] using the phyloseq and vegan packages.

## Results

### Replicability within our microcosm experiments

**Fig 1:**
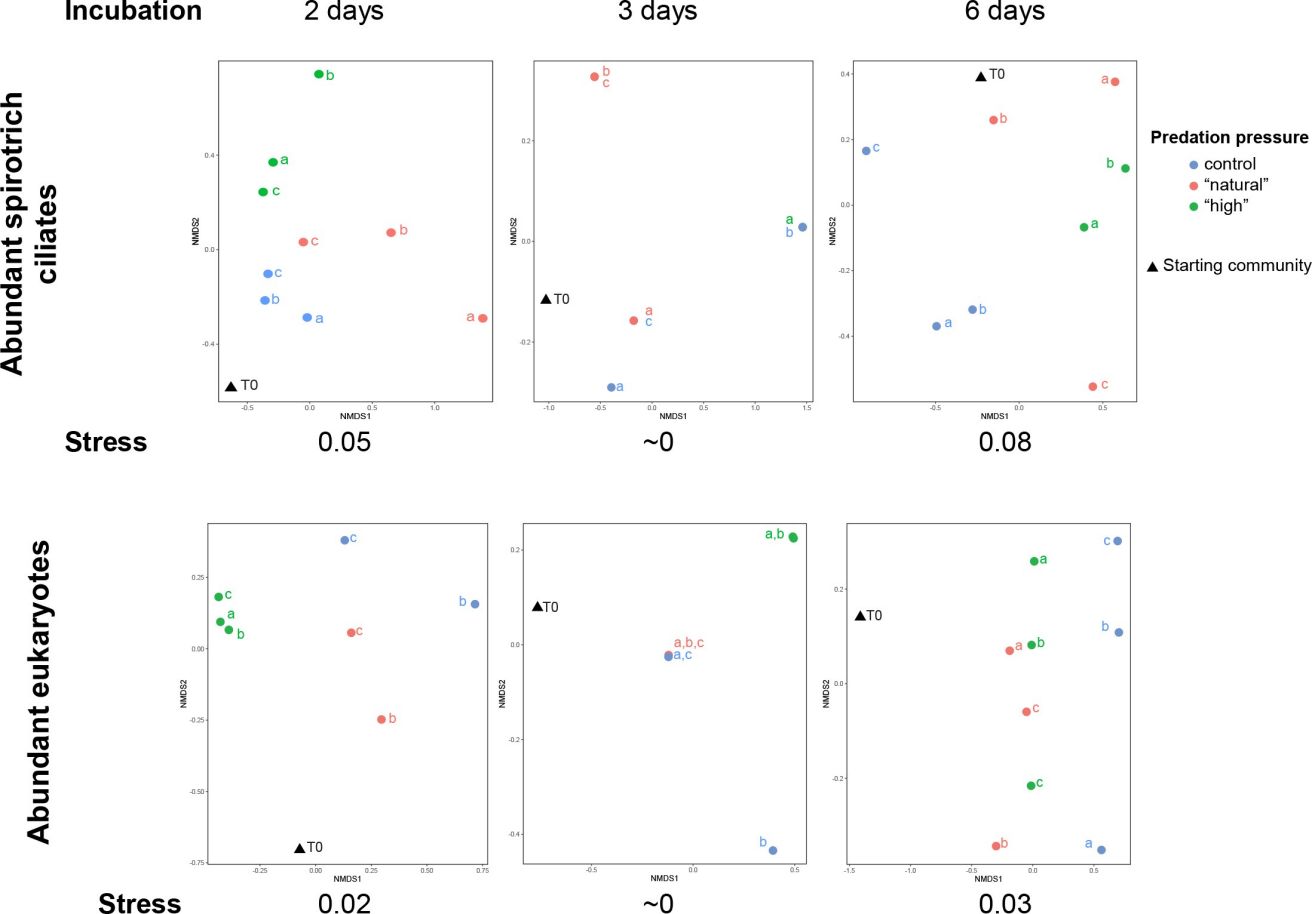
Non-metric multidimensional scaling of the DGGE band patterns using binary Jaccard index shows the good biological replicability of spirotrich and eukaryote communities when incubated for two days and more variability when incubated longer. a, b, and c represent the replicates, and T0 the starting community (before incubation). Control represent the community incubated without copepods added, ‘natural’ the natural predation pressure (10 copepods. L^-1^ added), and ‘high’ the high predation pressure (20 copepods. L^-1^ added). From left to right, the two, three, and six days incubations. The top panels are the abundant spirotrich ciliate communities and the bottom are all abundant microbial eukaryotes.

In the DGGE gels, which only capture abundant community members, replicates using both the spirotrich ciliate and all-eukaryote primers showed some variability but overall a similar response to the incubation (Fig 1 and S2 and S3 Tables and S1–S5 Figs). The number of bands using the spirotrich or all-eukaryotes primers ranged from 0 to 25 (average 8±4 and 11±5 bands per DGGE lane for spirotrich and all-eukaryotic primers, respectively). The number of bands (i.e. species) varied strongly within the biological replicates for each treatment and for both experiments (up to 8 times higher between two replicates, S2 and S3 Tables). The average number of species per treatment and the number of shared species show a significant correlation (Pearson correlation coefficient r = 0.85, P<<0.0001). This suggests that, while there is some variability within our biological replicates, the main pattern of response to a treatment is shared among replicates.

Abundant community members responded in the same way to the incubation in the two-day TD experiment, but biological replicates diverged when incubated longer (3 or 6 days). The brightest bands for the same treatment (i.e., C, N or H) are shared across replicates for both spirotrich and all-eukaryote primers in the two-day experiment (Figs 1 and S1). The longer (three and six days) TD experiments showed some variability (Fig 1), with the number of bands being up to 3 times higher (S2 Table), and the most abundant spirotrich species (brightest bands) were not shared among replicates (S1–S3 Figs).

Spirotrich communities in the BU experiments were more variable among replicates both in the number of bands and the identities of the brightest ones (S3 Table and S4–S6 Figs). In those experiments, even the replicates of the starting community contained some different abundant ciliate taxa (S4–S6 Figs). The number of shared taxa in the replicates ranged from 1 to 8. The starting communities contained from 10 to 20 ciliate bands in the top-down experiments, and from 5 to 17 abundant nanosized ciliates (2–10 µm) and 2 to 10 abundant microsized ciliates (10–80µm) for the BU experiments (top-down experiments were not size fractionated; S1–S6 Figs). Often, bright bands were common across 2 or 3 replicates (generally 1 or 2 highly abundant ciliates) but the less abundant taxa (fainter bands) showed less consistency (S1–S6 Figs).

### Spirotrichea ciliate community composition

We analyzed the diversity of Spirotrichea by sequencing 84 bands isolated from *DGGE spiro* (S1–S6 Figs and S4 Table). Six of these 84 bands BLASTed as non-ciliates (4 dinoflagellates and 2 stramenopiles), indicating that the great majority of our bands represented our target taxa. The remaining 78 sequences were 90% or greater in similarity to 30 spirotrich ciliate morphospecies deposited by name in GenBank (i.e. not “uncultured”). Given the low level of sequence similarity (90%), this is a conservative estimate of the total number of lineages in our experiments; i.e. these 30 taxa include genera as well as species and likely include intraspecific variability. We found some species present in almost all of our microcosm experiments. Specifically, we repeatedly found haplotypes related to *Pelagostrobilidium paraepacrum* FJ876963 (bands 6D-3, 3D-1 and 4D-2 in the top-down experiment, and Phyto26 and Phyto30 for the bottom-up experiment), *Eutintinnus tubulosus* JX101855 (e.g., 3D-2), *Rimostrombidium veniliae* FJ876964 (e.g., 2D-5 and Phyto43), and *Leegardiella* sp KY290313 (e.g., 3D-7 and Phyto14; S1–S7 Figs and S4 Table).

### Incubation effects

Incubation itself impacted the abundant members of the ciliate community as the numbers of bands tended to decrease during the course of the TD incubation (between the T_0_ and the controls at the end of the experiment; S1–S6 Figs). Between 3 and 8 bands disappeared during the TD incubation and for all but the shortest (2 days) experiment; only a few spirotrich haplotypes stayed dominant during the incubations (only 1–2 bright bands on final DGGE gels). In contrast, the most abundant (brightest bands) members of the eukaryotic assemblage did not show a strong change following incubation (S1–S3 Figs), indicating that dominant eukaryotes did not change during the TD incubation while spirotrich ciliates did.

Given that even the starting community was variable for the BU experiments, we focused on bands shared across at least two replicates and changes in the composition of the whole community (number of bands) across all replicates. For two of the three BU experiments, we observed a decrease in diversity over time, with 1–9 abundant ciliate bands of the nanosize fraction lost in our controls (BU2 and BU3, S3 Table). On the other hand, diversity of abundant spirotrich ciliates increased in the first experiments (BU1). In the microsize fraction, the pattern was more complex, with higher variability among the replicates (S3 Table), but the average number of bands was similar before and after incubation.

Some spirotrich haplotypes abundant in the starting community, specifically those related to *Pelagostrobilidium paraepacrum* (e.g., 3D-1), *Strombidinopsis sinicum* (e.g., 6D-1, 6D-2), *Strombidium paracapitatum* (e.g., 2D-6 and phyto35), *Rimostrombidium veniliae* (e.g., 6D-6), almost never remained abundant in our incubations (S1–S6 Figs). We also observed that some ‘rarer’ taxa in the starting community became more abundant in our controls (2D-7, 3D-2; S1–S6 Figs). Interestingly, all bands with BLAST hits closest to one or more *Eutintinnus* species (a loricate genus) in the top-down experiments (3D-2, 6D-5, 2D-10, 2D-8, 2D-9, and 2D-13) increased in controls relative to T_0_, but usually suffered more from grazing (decreased band brightness) in the treatments with the highest copepod abundance.

### Top-down experiments (DGGE Spiro *vs* DGGE alleuk)

Our DNA fingerprinting approach reveals that copepods impacted the diversity of abundant spirotrich ciliates while other abundant eukaryotes were not affected. Comparing the number of abundant spirotrich ciliates in the control and in our ‘natural’ predation pressure treatment (10 copepods), we observed a decrease in the number of bands when incubated for two days and a slight increase when incubated for three days (S2 Table). Intriguingly, for the high predation pressure treatment (20 copepods L^-1^), the number of bands was slightly greater than at the lower predation level for the two day incubation (experiment TD1) but distinctly lower for the three-day incubation (TD2; S2 Table). In sum, the number of abundant ciliates decreases with increasing predation pressure (i.e. number of copepods added) when incubated for three days (ANOVA, p<0.002), but not for two days (P>0.05). For the longest incubation (six days, TD3), the number of abundant species stayed nearly constant regardless of the number of copepods added (S2 Table), but the identity of the dominant spirotrich ciliates changed (Fig 1 and S2 Table and S4 Fig). No more than one band was common to all replicates, even in controls, underscoring the effects of the experimental manipulation itself on the assemblages during long incubations, and making it difficult to interpret this experiment.

In contrast to the ciliates, other dominant microbial eukaryotes did not change much in response to the addition of copepod grazers or the incubation itself. The abundant eukaryotes ranged from 10 to 25 species (i.e. DGGE bands) at the beginning of the experiments and did not change much across the treatments (S2 Table). The brightest bands (most abundant species) also did not change much with treatment or duration of the incubation (S1–S3 Figs). The number of species shared among all replicates stayed constant in number and composition whatever the number of copepods, and was relatively constant across experiments (S2 Table). In the three-day experiment (TD2), average number of bands decreases from 11.7 to 5.3 with increasing copepods (p<0.02; S2 Table), but this is skewed by the complete absence of bright bands in one replicate. We also observed that the number of abundant eukaryotes was not significantly related to the number of abundant spirotrich ciliates (regression analyses, P>0.05).

### Bottom-up experiment—impact on Spirotrichea (DGGE vs HTS)

**Fig 2:**
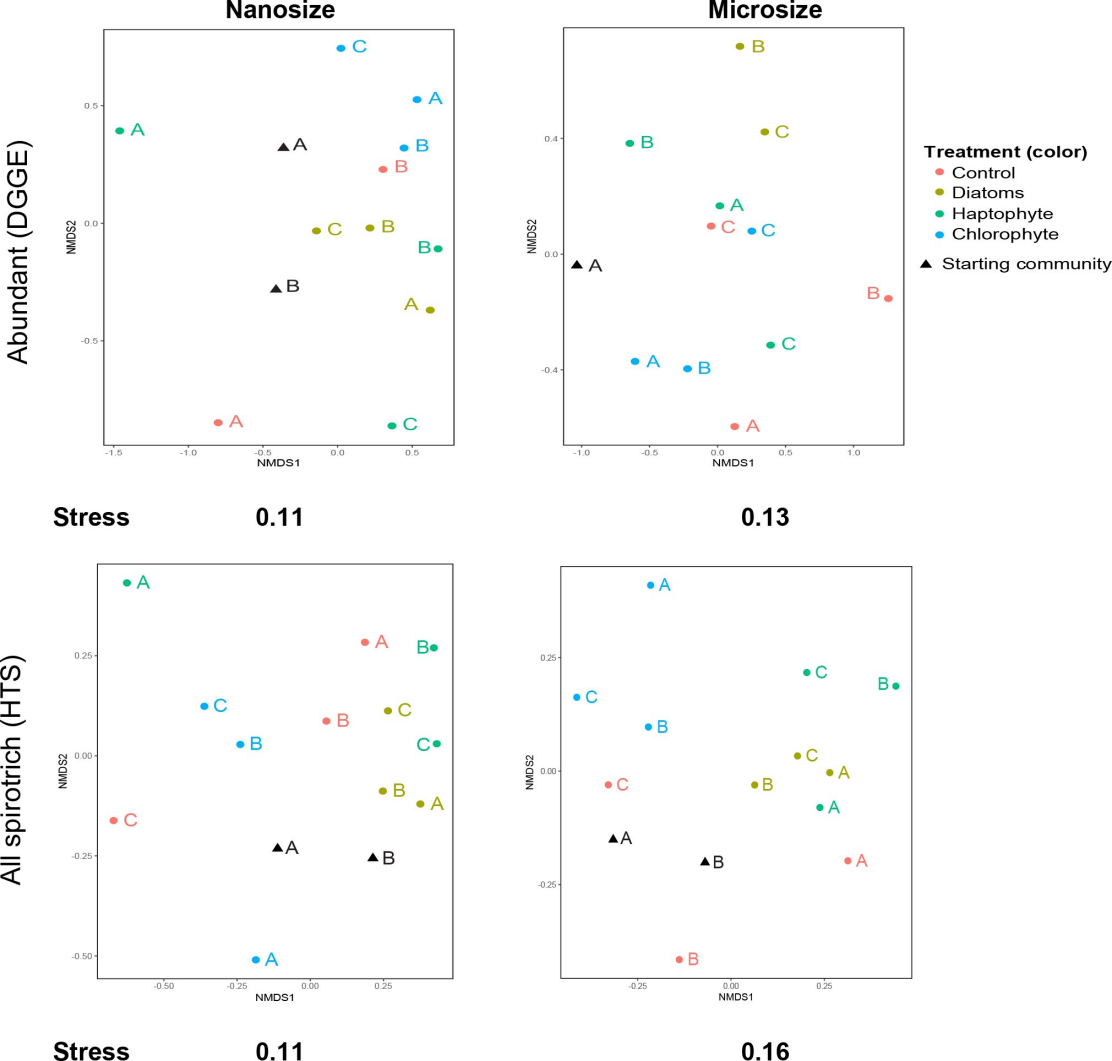
Non-metric multidimensional scaling using Jaccard binary index of the spirotrich community shows the considerable variability among biological replicates when communities are incubated with three different kinds of phytoplankton. A, B, and C represent the replicates. ‘Starting community’ is the community before incubation. ‘Control’ represents the community incubated without perturbation. ‘Diatoms’, ‘Haptophyte’ and ‘Chlorophyte’ represent the incubation with addition of phytoplankton. Left is the nanosize and right the microsize fraction. The top panels are the abundant spirotrich ciliates assessed by DGGE and the bottom are the spirotrich ciliates assessed by HTS.

The bottom-up experiments (BU) show variable responses of abundant ciliates to simulated blooms of various kind of phytoplankton. For experiment BU1, the abundant nanosized spirotrich richness increases after three days incubation for all treatments and the control (S3 Table). We observe the inverse trend for BU2 and BU3 i.e. decrease of nanosized species richness (S3 Table). In contrast, the abundant microsized spirotrich richness did not change across replicates, treatments and experiments (i.e. in BU1, 2 and 3; S3 Table). Abundant nanosized and microsized spirotrich ciliates thus did not respond clearly to our bloom treatment. The NMDS shows some grouping for the nanosized community (e.g., chlorophyte treatment) and for the abundant microsized spirotrich (e.g. diatom treatment), but the groupings are not significant (Fig 2).

To have a better understanding of the impact of blooms on the spirotrich community we used SAR primers for HTS amplicon sequencing. Analyses of HTS data found that OTU richness varies from 92 to 148 spirotrich species per treatment, with a slightly higher richness for the community incubated with the chlorophyte (148) and slightly lower with the diatom (92). The distribution of species richness by DGGE and HTS did not match, likely due to the different methods and primers used. However, as for the DGGE, the community composition did not seem to be related to the phytoplankton (Fig 2). Indeed, communities incubated with the same phytoplankton species are close but are still overlapping with those in the other treatments (Fig 2).

### Bottom-up experiment—impact on the SAR clade (HTS)

**Fig 3:**
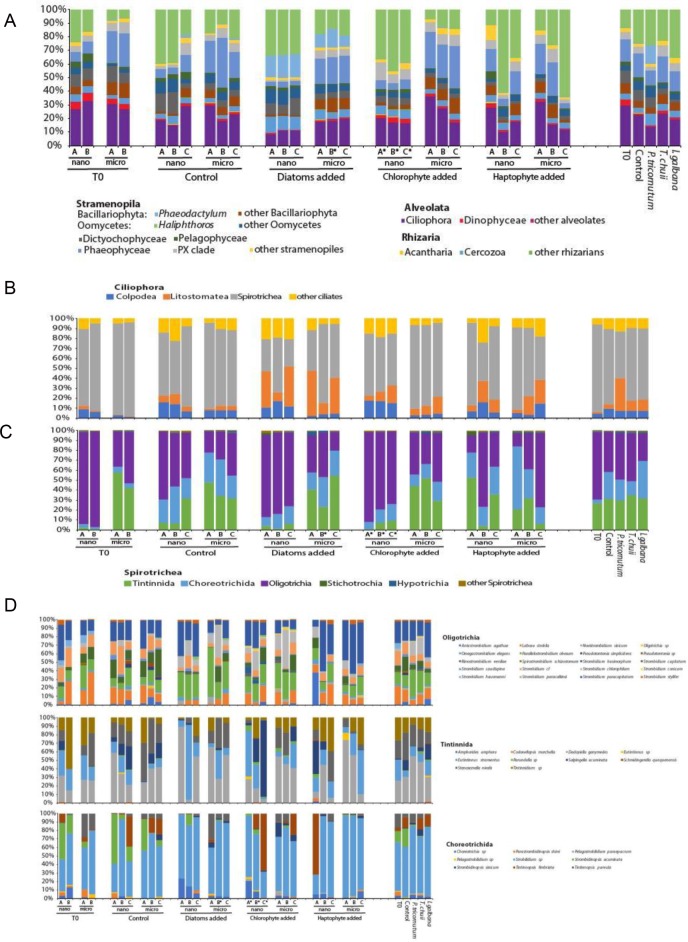
**Distribution of dominant taxa in our phytoplankton microcosm experiment analyzed using high throughput sequencing (HTS) with SAR-specific primers: (A) dominant SAR lineages (B) ciliates classes, (C) spirotrich orders and (D) species.** For each panel, the left part of the bar graph shows replicates for each size fraction and for each incubation condition, while the right part represents only treatments (i.e. replicates and size fractions have been pooled). The * denotes samples with fewer than 60,000 reads (see methods).

In contrast to the variability in response of spirotrich ciliates to the phytoplankton treatments, analyses of HTS data indicate a consistent SAR community among replicates for each treatment, except for the haptophyte addition (Fig 3B–3D). Perhaps surprisingly, the initial stramenopile assemblage was dominated by oomycetes, with that group representing about 1/3 of all OTUs in the 2–10 µm size fraction (Figs 3A and S8). OTU4, closely-related to the marine oomycete *Haliphthoros* (pairwise distance of 0.09), was the most abundant. Overall, the two size fractions were dominated by different SAR clades: Phaeophyceae (brown algae) and Bacillariophyceae (diatoms) in the microsize fraction (10–80µm), and oomycetes (mainly due to *Haliphthoros* a parasite of marine arthropods [65]) and Cercozoa in the nanosize fraction (2–10µm; Fig 3).

**Fig 4:**
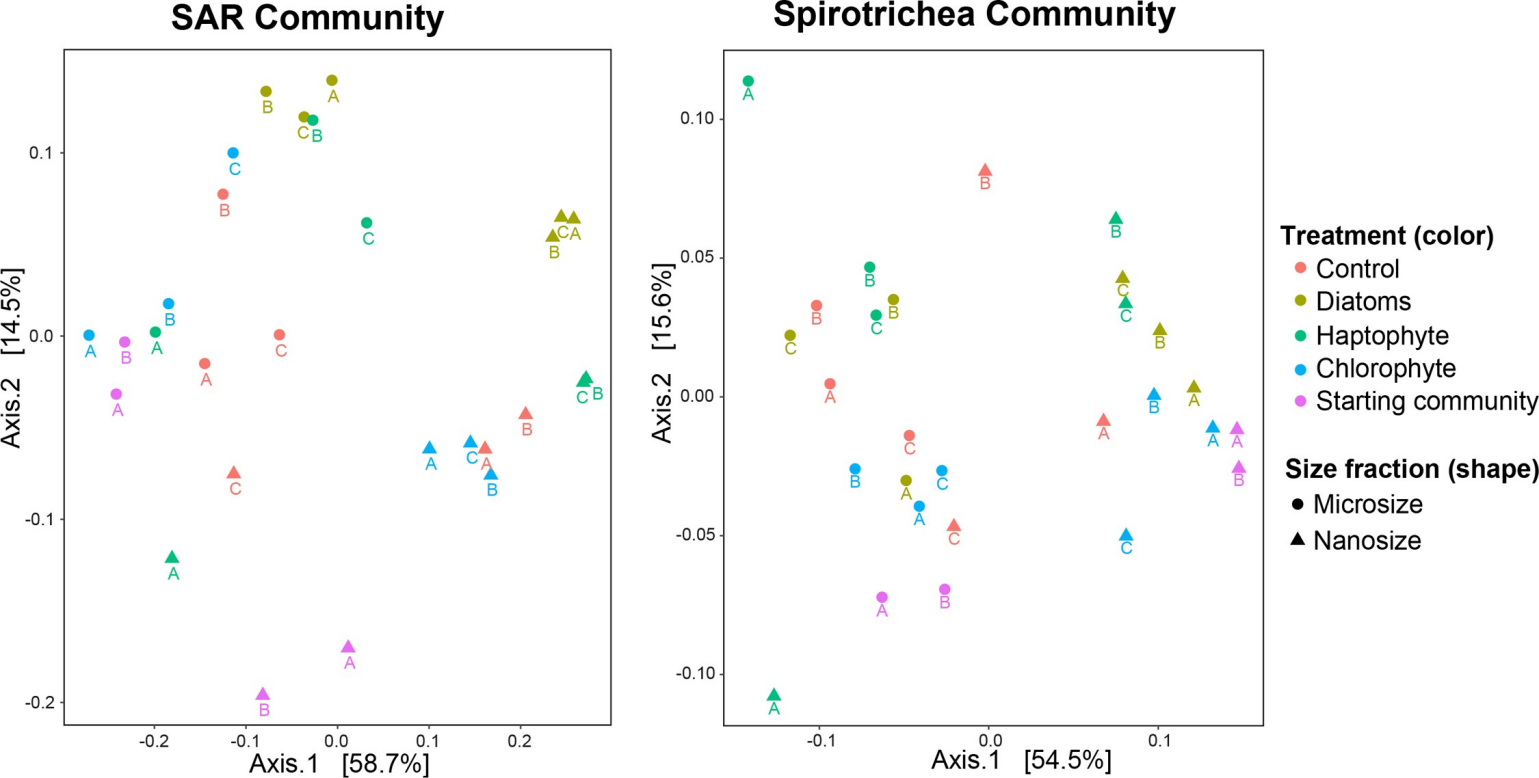
**Principal Coordinate analyses of HTS data show (1) clustering of distinct SAR and Spirotrichea communities that are related to size fractions (circle and triangle) and (2) a tendency to group by phytoplankton treatments for the SAR community (3) but not for the ciliate community.** The Unifrac dissimilarity index has been computed for these analyses. The same analyses were carried out with the Bray-Curtis index and gave the same pattern.

Using principal coordinate analysis and the Unifrac dissimilarity index, the SAR assemblages cluster by size fraction (PERMANOVA R^2^ = 0.35 P<0.001) such as the spirotrich ciliates (R^2^ = 0.26 P<0.001; Fig 4 and S6 Table). Overall, the SAR and Spirotrichea communities, nanosize, microsize or both fractions, are significantly impacted by the experiment (Treatments in S6 Table). Among treatments, the diatom addition experiments cluster tightly, perhaps driven in part by the fact that the added diatom is itself a member of SAR. The chlorophyte addition treatments cluster only in the 10–80 µm fraction, while the haptophyte additions and the controls do not cluster at all (Fig 4). None of the phytoplankton treatments (‘Diatoms’, ‘Haptophyte’ and ‘Chlorophyte’) show a significant difference compared to each other (pairwise comparison with Bonferroni correction P>0.05), suggesting (1) a minimal effect of species used to simulate the phytoplankton bloom on SAR and Spirotrichea communities, or (2) the dominant species are not impacted by the phytoplankton we added. Because the initial assemblages in both size fractions cluster significantly, we conclude that the SAR assemblages in both the nano- and micro-sized fractions diverged over time (Fig 4 and S6 Table).

## Discussion

### Biological variability of spirotrich ciliates in microcosm experiments

Our spirotrich ciliate communities showed strong variability when incubated longer than two days, with variation between replicates being higher than variation between treatments. While our biological replicates showed variability, the technical replicates in our DGGE analyses showed the same community composition, suggesting that incubation itself caused the communities to diverge. The PCR and DGGE have been carried out multiple times to make sure that the variability observed is not related to a technical issue. These procedures are highly repeatable. The variability within the Spirotrichea in the long incubation may be resource related (i.e. the microcosms diverged due to stochastic effects as food resources became more limited; Fig 1) and in the three day incubation due to low starting diversity (TD2, maximum of 8 abundant species; S2 Table). Indeed, ciliates of mid-latitudes generally show highest abundance in late spring—summer and lower abundances late fall—winter [16, 66, 67]. The low abundances in these November experiments may have led to the strong variability in our DGGE results, particularly if rarer taxa that were distributed unequally between replicates increased during the incubations.

Another factor impacting our biological replicates is the variability in the starting community of the bottom-up experiment, which is intriguing. The species richness (number of bands) and the community composition (band pattern) of the *in situ* community replicates were inconsistent (species richness varying by up a factor 8, and few bands in common; S3 Table; S4–S6 Figs). The possibility of community variation at small and fine scales is not new and has been reported elsewhere [68]. For example, Dolan and Stoeck [69] show dissimilarity between true replicates ranging from 4 to 12% looking at ciliates by morphology. Using molecular tools, Lie et al [68] showed only 71% similarity among true replicates by T-RFLP in samples from the coastal North Pacific Ocean.

The presence of abundant taxa in our experiments that have also been found in other North Atlantic studies suggests that our microcosm experiments are representative of the *in situ* community in the New England. The diversity observed in our experiments is similar to previous observations: 8–26 common OTUs in Long Island Sound [47, 70], between 2 and 22 on the nearby shelf by DGGE [53,54] and between 1 and 67 using HTS [54,71]. While the number of species obtained by HTS is higher in the present study compared to this earlier work, we should note the difference in primers (here we considered all spirotrich and not only oligotrich and choreotrich) and that the HTS methods were different (454 vs MiSeq, which leads up to 100 times more sequences, so potentially rarer OTUs). However, the identity of spirotrich ciliates observed in our microcosms is similar to the ciliates observed *in situ* in the same area (S7 Fig, [47,54,70]). Some ciliates observed in all DGGE gels (e.g., *Pelagostrobilidium paraepacrum*, *Eutintinnus tubulosus*, *Rimostrombidium veniliae*, and *Leegardiella* sp) were also observed during a tide pool survey in the same area during the summer 2015 [72], in Long Island Sound [47, 49, 50, 73], in the nearshore area during the summer 2014 [55], and as members of an assemblage present from the nearshore to beyond the shelf break of New England across multiple depths during summer 2012 [53, 71]. The consistent abundance of these OTUs suggests they play key ecological roles in North Atlantic waters or are opportunistic species. Also, Capriulo et al [74], in a three-year study of Long Island Sound, documented by microscopy a total of 67 spirotrich ciliate morphospecies, so we conclude that a substantial amount of the abundant spirotrich ciliate diversity was included in our microcosms (78 spirotrich haplotypes).

Looking at the SAR community, only one study has been published with this primer set in freshwater environments, and reported between 205 and 757 OTUs [48]. Rhizaria are underrepresented in the present study as in the freshwater study [48] compared to recent studies in marine environments [38]. This discrepancy is likely related to (1) our size fractionation and (2) to a primer bias. While Rhizaria has been regarded as one of the most abundant clades in ocean, the organisms are generally larger than 100µm [75] and so may have been eliminated by our 80µm prescreening step. Also, one of the major contributors to the Rhizaria are the Foraminifera, which have a complex SSU rRNA gene that would not be amplified by our primer set.

### Incubation impacted abundant species

Experimental incubation is a fundamental tool in biology to assess physiological rates in microbial communities (e.g. primary and bacterial production, microbial growth and feeding experiments), but few studies have looked at the impact of incubation on microbial eukaryotes. In a study using Clone libraries and T-RFLP, a microbial community that was incubated for three days showed a similar impact on diversity over time, compared to our experiments, with only 18% of the species shared by the three time points and 65% of the species observed only at one time point [76]. In our case, the diversity of abundant species (DGGE bands) decreased during the incubation. Indeed, the number of ciliates bands in our starting community was almost always greater than the number of bands observed after incubation (S2 and S3 Tables). While each treatment are statistically different, HTS did not indicated differences between the starting community and controls (S6 Table). Four decades ago, a microcosm study based on morphospecies found that the ciliates were the group of microzooplankton most negatively impacted during 8- and 24-hour incubations [77].

The variability in our replicates makes it difficult to identify species that responded to the treatments, but there were some species that responded consistently across replicates. For example, a DGGE band related to the common *Strombidium paraepacrum* never remained abundant in the mesocosms whatever the time of the year or the duration of the incubation. Another band related to *Eutintinnus* usually increased during the incubations, suggesting amenability to confinement in the mesocosms. Alteration of the community composition incubated under similar conditions has been reported elsewhere [76, 78]. This suggests that the incubation itself can select for one or a few taxa well-adapted to experimental manipulation, consistent with the fact that only a small fraction of ocean microbes are cultivable [79]; some opportunistic species (e.g. *Eutintinnus*) may benefit from reduction in predation and turbulence or other features of the dialysis bag environment.

Changes in community composition under incubation complicate evaluation of the representativeness of our measurements. Other studies have also reported incubation-induced changes in microbial communities [80], which can also impact grazers and other parts of the community [81]. Many hypotheses have been proposed to explain this often-observed phenomenon. For example, this “bottle effect” may be due to the impact of surfaces in contact with the microbial community under confinement. Another explanation is derived from the famous “paradox of the plankton”–existence of high diversity in a relatively homogeneous environment [82]. One resolution to this paradox is that natural plankton populations are not in equilibrium because continual disturbances prevent competitive exclusion. Given high diversity and a limited number of niches [82], functionally redundant species in the plankton can replace each other randomly under constant disturbance from mixing, advection, etc., across small spatial distances. In our experiments, the communities come from the same metapopulation and were incubated under the same conditions (niches), so we expected to observe the same resulting community (given that the same competitive interactions occurred); this is not what we found. For the same niches and from the same metapopulation, minus the continual disturbances, different communities emerged after confinement. We speculate that each mesocosm replicate may represent a small sub-community where small initial differences put them on different trajectories of assembly from a highly diverse metacommunity. In the absence of disturbance, the mesocosms will continue on their different trajectories and diverge from their initial composition, irrespective of treatment.

### Top-down experiments–Copepods prefer ciliates

In the shorter copepod experiments (two and three days, TD 1 and 2), the number of abundant ciliates (DGGE bands) decreased with the presence of copepods, while the number of abundant total eukaryotes (*DGGE alleuk*) did not decrease (S2 Table and S1 and S2 Figs). Calbet and Saiz [27] have shown that ciliates are an important component of copepod diets and can represent up to 50% of their carbon consumption. This leads us to conclude that results of our copepod experiments support the preference of copepods for ciliates, compared to many of the other eukaryote prey items available.

For the six days incubation experiment (TD 3), we observed stable ciliate diversity with increase in the number of adult copepods added (S2 Table). This may be related to cannibalism by copepods as adult copepods can consume up to 35% of available nauplii per day [83], suggesting that added copepods may have been consuming primarily nauplii instead of grazing on the ciliates in the 6d incubation. Depending on the relative abundance of different foods, many copepod species switch feeding modes from picking small particles out of a feeding current to raptorial ingestion of nauplii [84, 85]. Thus, the ciliate diversity remaining similar in the copepod treatments may be related to *lower* predation pressure as copepods ate the predators of the ciliates (nauplii).

One confounding issue in our top-down experiments is that the conditions in our microcosms allowed copepods to develop from nauplii to adults. We endeavored to enumerate the copepods in two of the three top-down incubations by screening bag contents at the end of the experiments (TD2 and TD3; S5 Table). As with the micro- eukaryotes, we observed variability in copepod abundance within biological replicates. Indeed, copepods grew in some of the bags, and we observed between 12 and 27 adult copepods in our control bags at the end of the six day incubation, and from 2 to 11 in the two days incubation. Our prescreening with a 200µm mesh at T_0_ removed adult copepods but not copepod nauplii (c. 60–80 µm), which have the time to grow up, particularly in the samples incubated for six days. For example, the copepod abundance was greater in the control than in the copepod treatment by the end of the six days incubations (S3 Table) and was higher in our ‘natural’ predation than in our high predation pressure treatment. This suggests that adult copepods have a negative effect on juvenile copepod growth.

The fact that some of the copepod nauplii in the control microcosms had the time to develop to copepodites illustrates the difficulty in interpreting results of experiments on complex food webs in which life stage transitions may result in changes in trophic links during incubation. At present, the impact of copepod juveniles (nauplii and copepodites) on ciliates is not well known [86]. In our experiments, the main impacts appeared to be due to the added adult copepods, but further experimental approaches to this issue are warranted.

### Bottom-up experiment—a stochastic effect on Spirotrichea

The three bottom-up experiments showed variable responses of abundant ciliates to increasing phytoplankton. The non-replicability may be due to the variability in starting community members from the fall sampling, as discussed above. Besides the lower abundance of ciliates in the fall, the lack of response to the phytoplankton treatments may have also resulted from the ciliates already being food-saturated or feeding on particles other than phytoplankton [17, 87–89].

To explore further the variability in the third bottom-up experiment (BU3), we used amplicon high-throughput sequencing (HTS) with primers designed to capture the diversity of the whole SAR (Stramenopila, Alveolata, and Rhizaria) clade. Unlike DGGE, HTS samples the community comprehensively, revealing even very rare members. We first focused on the diversity of ciliates revealed by our HTS analyses of experiment 3 and found that the ciliate assemblage was dominated by the class Spirotrichea (and subclasses Choreotrichia and Oligotrichia) as previously documented (S6B and S6C Fig; [90–93]). Looking at the whole SAR community, we observe an increase of litostome ciliates and oomycetes in all phytoplankton treatments relative to the initials and controls, particularly in the diatom treatment. This likely represents the increase in opportunistic “weed” species that prosper under reduced grazing, eutrophication, and confinement. While we observed a significative impact of the phytoplankton bloom on SAR and Spirotrichea communities, none of the three phytoplankton species results in a different response of the *in situ* community. This suggests that only prey size matters in the selection of food by microheterotrophs, as the three phytoplankton species used have the same size range [87–89].

## Synthesis

Although there was variability in the responses to the treatments in our various microcosms, some broad conclusions can be made. Overall, we found that ciliates were preferentially grazed by copepods, compared to other eukaryotes. The lack of clear reproducibility within our biological replicates suggest that the community was heterogeneous at very small scales (the size of our bag was around 500mL) or that the community responded ‘randomly’ to confinement. While surprising, this is in agreement with previous *in situ* observations failing to clearly relate spirotrich [54, 55, 71] and microbial eukaryote diversity [94, 95] to environmental parameters.

Dialysis bag microcosms allow fully replicated, multi-variable experiments to be performed with microbial communities under simulated *in situ* conditions. To improve reliability, we recommend a higher density of organisms and an increase in the number of replicates. Despite inter-replicate variability, these kinds of experiments can help elucidate factors controlling microbial eukaryote diversity, growth and ecological roles in the plankton.

## Supporting information

S1 Fig DGGE of two-day top down control experiment (TD 1) top: spirotrich ciliate primers; Bottom: All-eukaryote primers) reveals similar responses (band patterns) among replicates. Std are standard used to compared DGGE gels, T0 the starting community, C the controls, N the ‘natural’ predation pressure samples, and H the ‘High’ predation pressure samples. a, b, and c represent the replicates.
(DOCX)

S2 Fig DGGE of three-day top down experiment (TD 2) reveals similar responses among replicates using spirotrich ciliate or eukaryote primers (top and bottom pictures, respectively). Other notes as in S1 Fig.
(DOCX)

S3 Fig DGGE of six-day top down experiment (TD 3) reveals variable responses of the dominant spirotrich ciliates and similar responses of the dominant eukaryotes among replicates. Other notes as in S1 Fig.
(DOCX)

S4 Fig DGGE of bottom-up experiment 1 (BU 1) using Spirotrichea primers shows high variability among replicates. Each lane presents a replicate of T_0_, control, and three bloom treatments. T_0_ has two replicates (A-B) and the other treatments have three replicates (A-C). Brightness of bands indicates how abundant a taxon was within its community. Red numbers represent bands that were sequenced.
(DOCX)

S5 Fig DGGE of bottom up Experiment 2 (BU 2) using Spirotrichea primers shows high variability among replicates. Other notes as in S4 Fig.
(DOCX)

S6 Fig DGGE of bottom up experiment 3 (BU 3) using Spirotrichea primers shows high variability among replicates. Each lane presents a replicate of Time Zero, control, and three bloom treatments. Other notes as in S4 Fig.
(DOCX)

S7 Fig Phylogeny of the DGGE haplotypes (orange) reveals diversity of lineages generated using spirotrich ciliate DGGE primers. The reference morphospecies are in red for Tintinnida, in green for Choreotrichida and in blue for Oligotrichia. In grey are previously sequenced DGGE haplotype [54,55,70,72] and in black are outgroup morphospecies. Sequences were aligned with Muscle and tree was built using the GRTGAMMAI parameter in RaxML.
(DOCX)

S8 Fig **Distribution of dominant (A) stramenopiles in our phytoplankton microcosm experiment analyzed using high throughput sequencing and SAR-specific primers.** The left part of the graph shows each replicate for each size fraction and for each incubation condition, while the right part represents only the incubations conditions (replicates and size have been pooled together). The * denote samples with less than 50,000 rarefied reads.
(DOCX)

S1 Table Primer sets used in this study. (DOCX)

S2 Table Number of abundant spirotrich (top) and eukaryotic (bottom) species (bands) in each replicate during the top down experiments. (DOCX)

S3 Table Number of abundant nanosize (top) and microsize (bottom) spirotrich ciliate species (band) during the bottom-up experiments. (DOCX)

S4 Table Top blast hits for the copepod and phytoplankton microcosm experiments. (DOCX)

S5 Table Copepod abundance during the copepod experiments. (DOCX)

S6 Table PERMANOVA results (R^2^) from HTS SAR data with 999 permutations. (DOCX)
