# Supplementary material for: Incubation and grazing effects on spirotrich ciliate diversity inferred from molecular analyses of microcosm experiments
Source: PLoS One. 2019 May 6;14(5):e0215872. doi: 10.1371/journal.pone.0215872 (PMC6502329; doi:10.1371/journal.pone.0215872)
Supplement: S1 Fig — Std are standard used to compared DGGE gels, T0 the starting community, C the controls, N the ‘natural’ predation pressure samples, and H the ‘High’ predation pressure samples. a, b, and c represent the replicates. (DOCX) [file pone.0215872.s001.docx]

**Std T0 C-a C-b C-c N-a N-b N-c H-a H-b H-c Std**

**
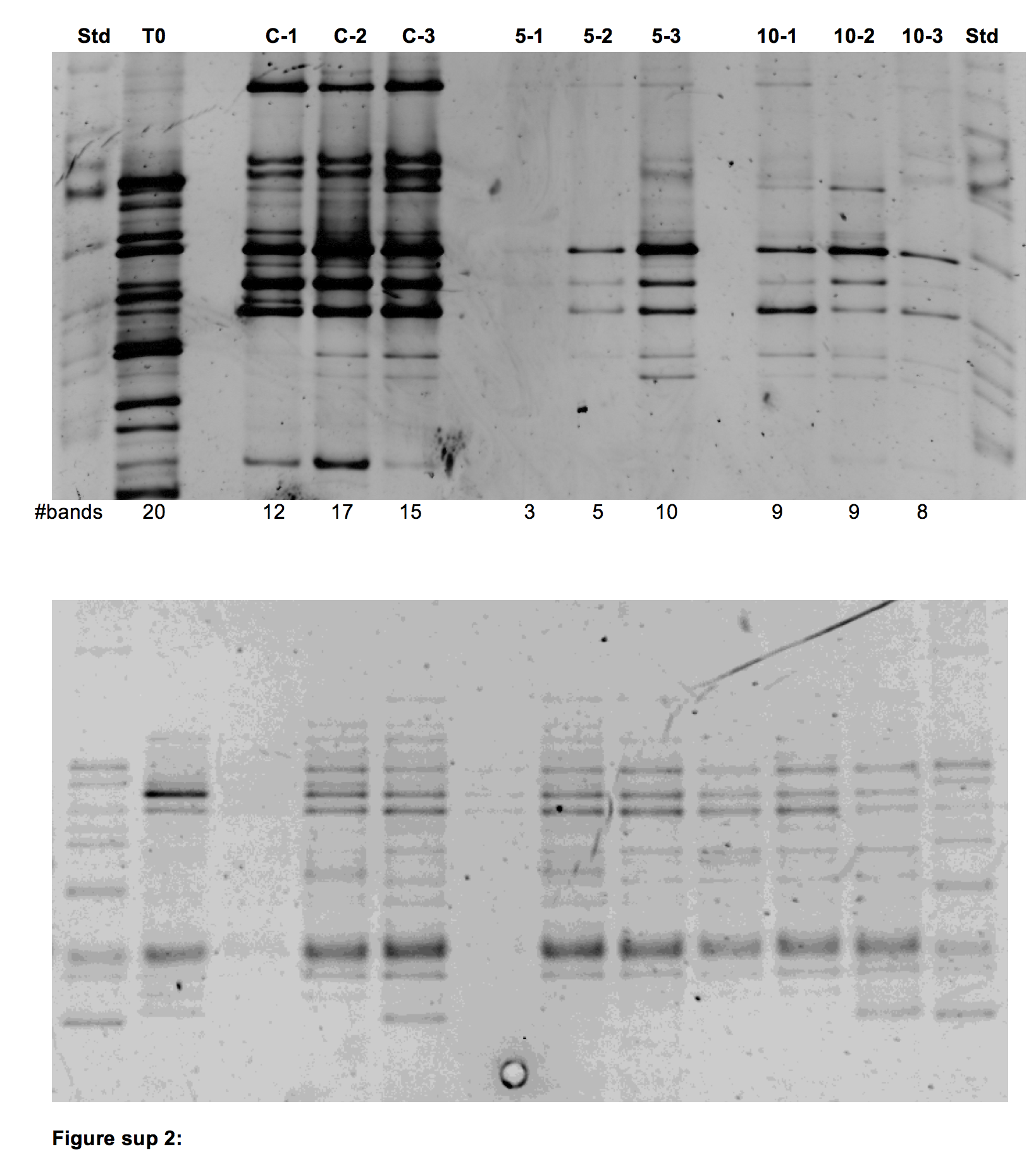
**

**Std T0 C-a C-b C-c N-a N-b N-c H-a H-b H-c Std**

**
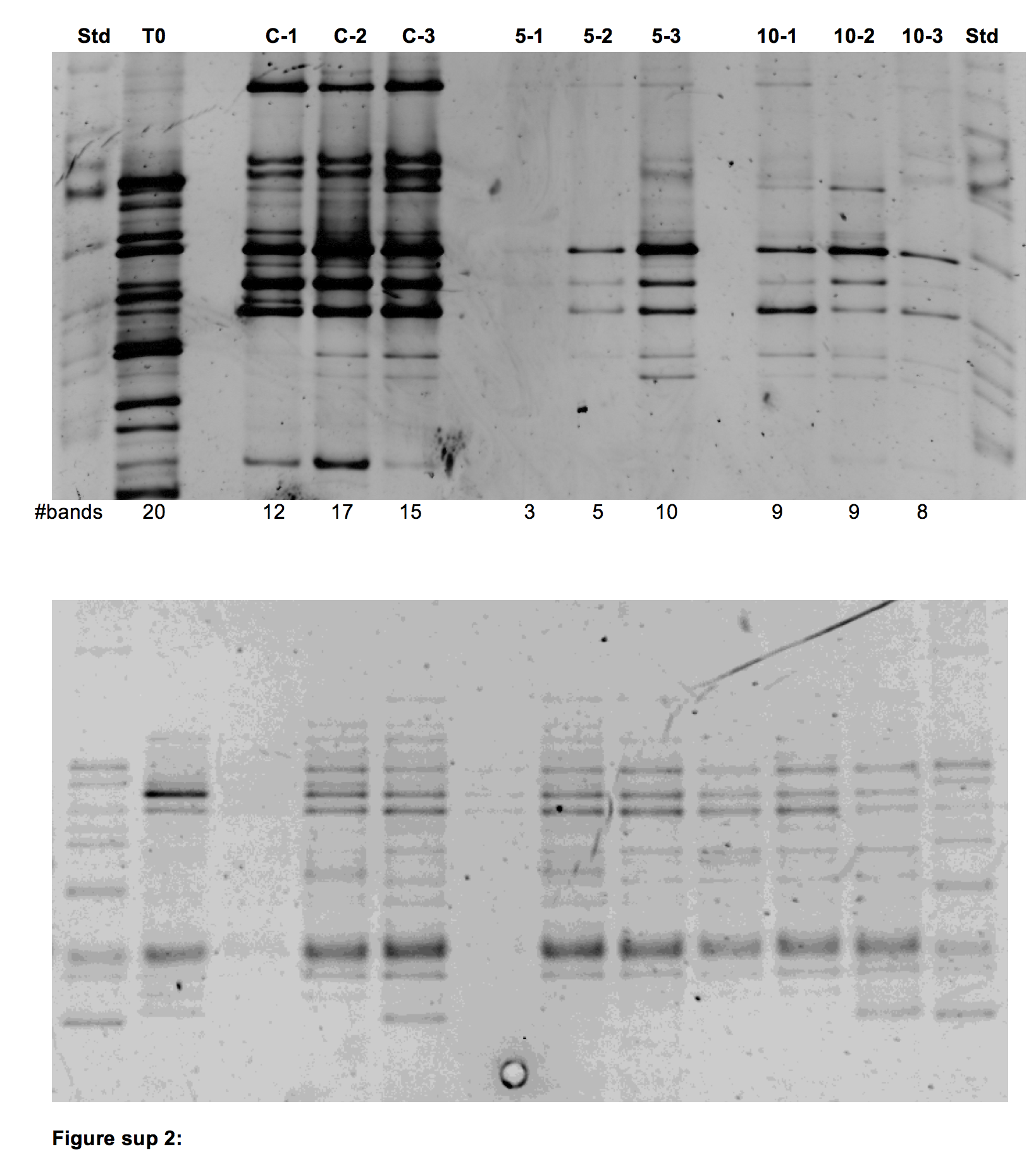
**

**S1 Fig. DGGE of two-day top down control experiment (TD 1) top: spirotrich ciliate primers; bottom: all-eukaryote primers) reveals similar responses (band patterns) among replicates.** Std are standard used to compared DGGE gels, T0 the starting community, C the controls, N the ‘natural’ predation pressure samples, and H the ‘High’ predation pressure samples. a, b, and c represent the replicates.
