# Supplementary material for: Incubation and grazing effects on spirotrich ciliate diversity inferred from molecular analyses of microcosm experiments
Source: PLoS One. 2019 May 6;14(5):e0215872. doi: 10.1371/journal.pone.0215872 (PMC6502329; doi:10.1371/journal.pone.0215872)
Supplement: S2 Fig — Other notes as in S1 Fig. (DOCX) [file pone.0215872.s002.docx]

**Std T0 C-a C-b C-c N-a N-b N-c H-a H-b H-c Std**

**
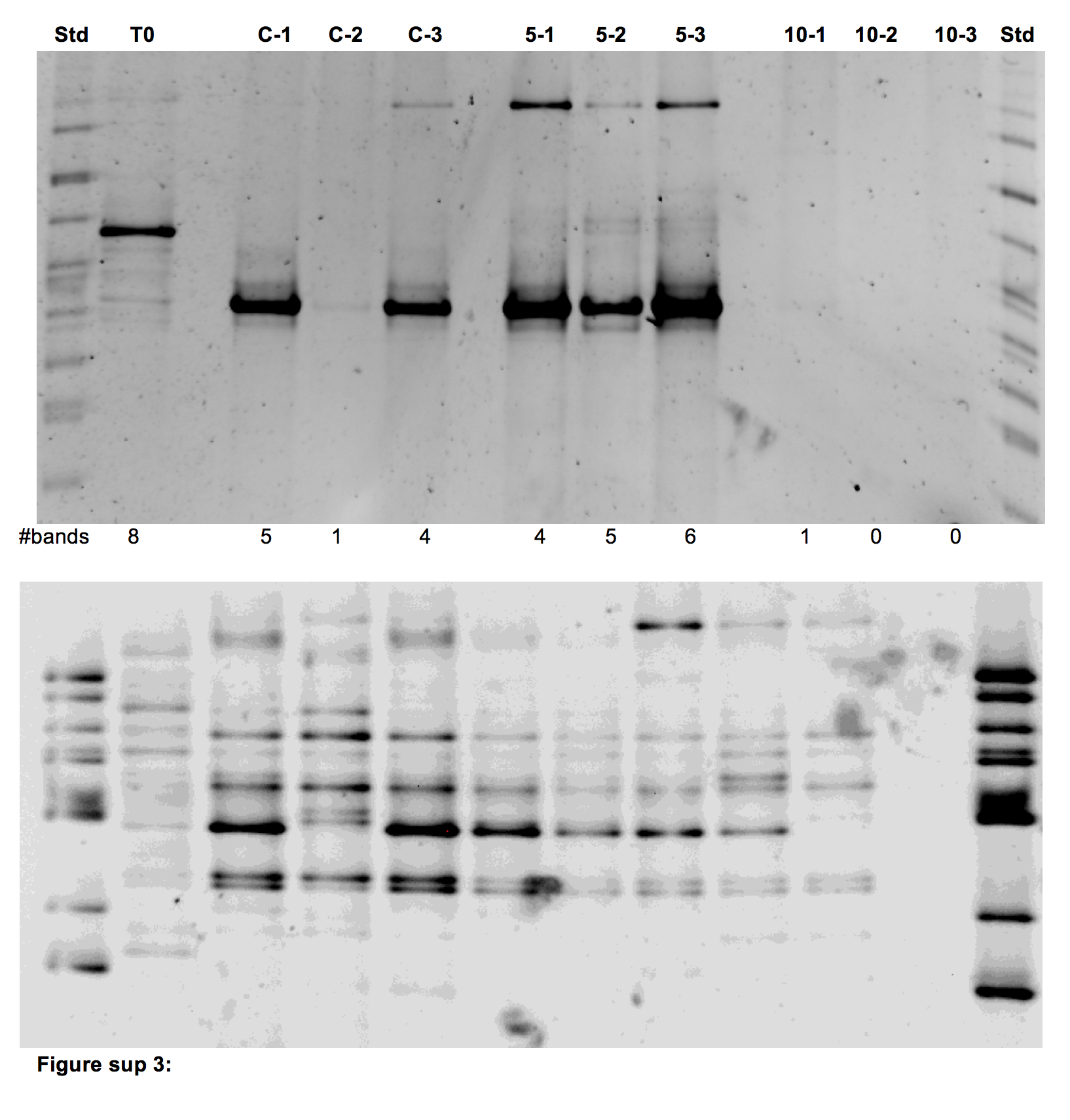
**

**Std T0 C-a Cb C-c N-a N-b N-c H-a H-b H-c Std**

**
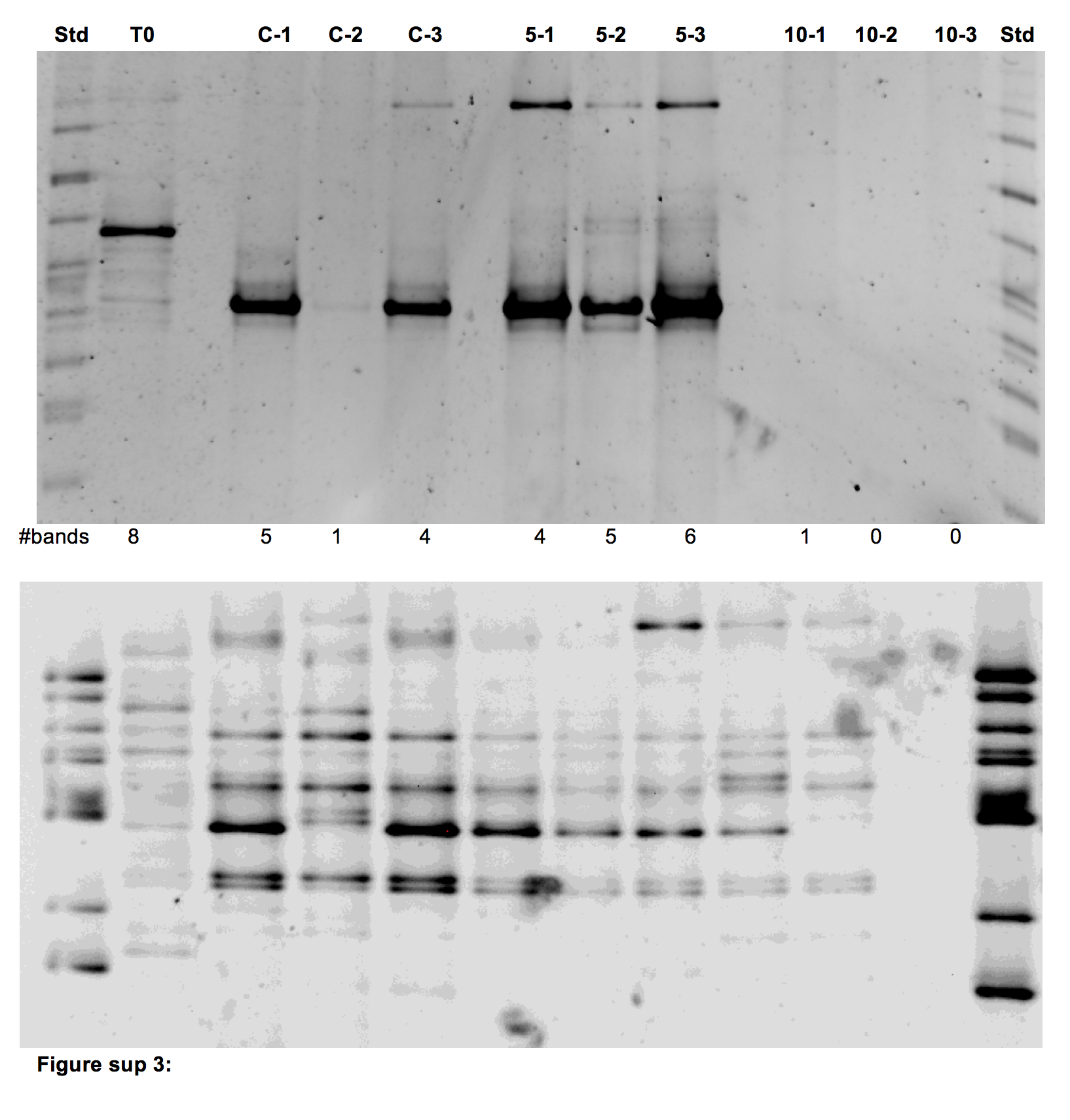
S2 Fig. DGGE of three-day top down experiment (TD 2) reveals similar responses among replicates using spirotrich ciliate or eukaryote primers (top and bottom pictures, respectively).** Other notes as in Figure S1.
