# Supplementary material for: Incubation and grazing effects on spirotrich ciliate diversity inferred from molecular analyses of microcosm experiments
Source: PLoS One. 2019 May 6;14(5):e0215872. doi: 10.1371/journal.pone.0215872 (PMC6502329; doi:10.1371/journal.pone.0215872)
Supplement: S3 Fig — Other notes as in S1 Fig. (DOCX) [file pone.0215872.s003.docx]

**Std T0 C-a C-b C-c N-a N-b N-c H-a H-b Std**

**
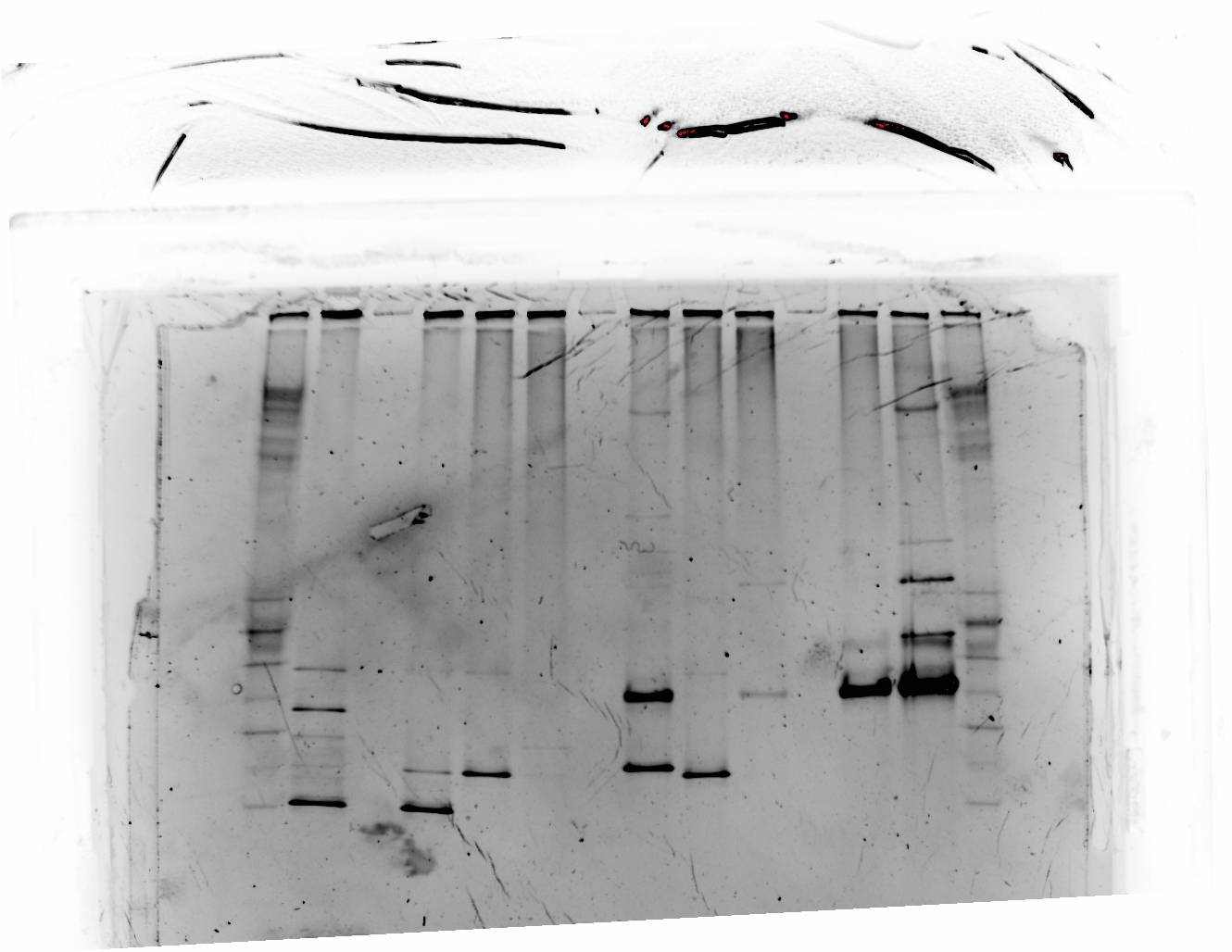
**

**Std T0 C-a C-b C-c N-a N-b N-c H-a H-b H-c Std**


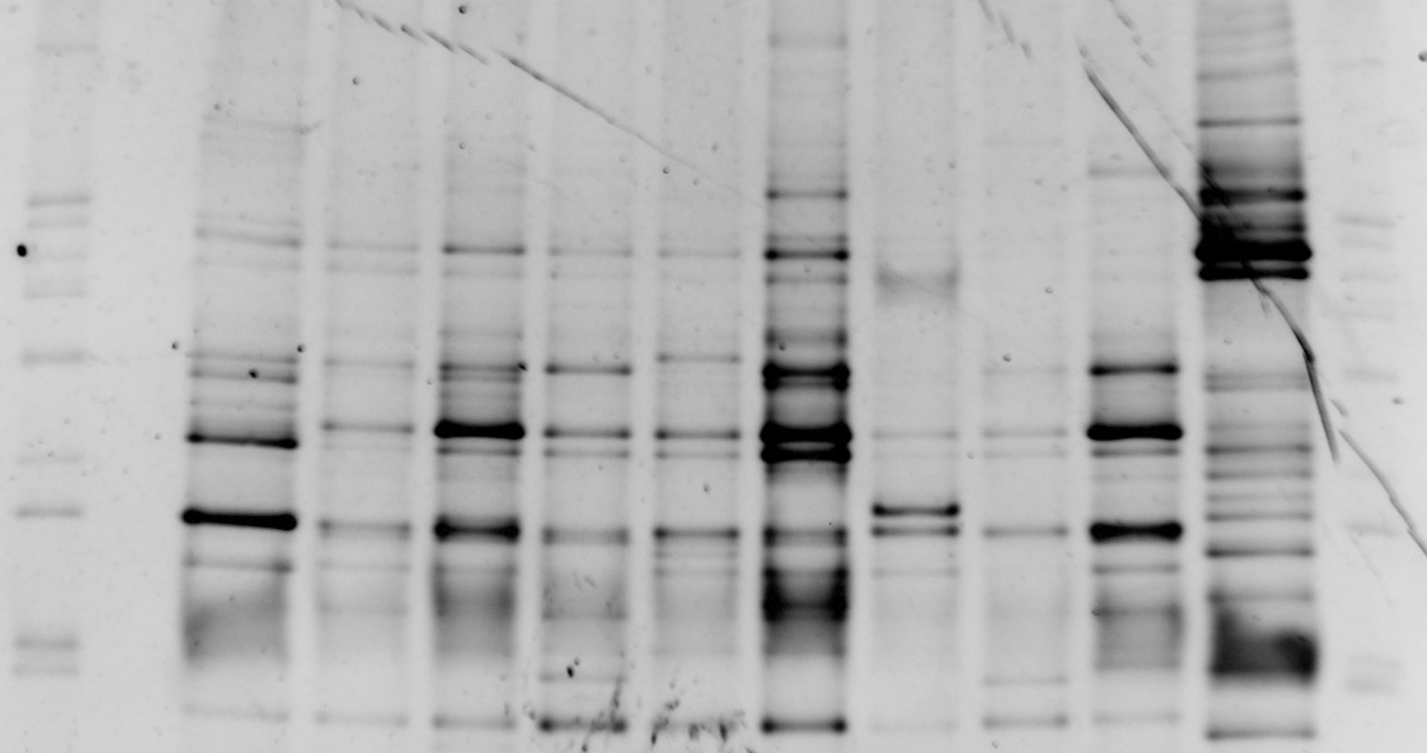


**S3 Fig. DGGE of six-day top down experiment (TD 3) reveals variable responses of the dominant spirotrich ciliates and similar responses of the dominant eukaryotes among replicates.** Other notes as in Figure S1.
