# Supplementary material for: Incubation and grazing effects on spirotrich ciliate diversity inferred from molecular analyses of microcosm experiments
Source: PLoS One. 2019 May 6;14(5):e0215872. doi: 10.1371/journal.pone.0215872 (PMC6502329; doi:10.1371/journal.pone.0215872)
Supplement: S4 Fig — Each lane presents a replicate of T0, control, and three bloom treatments. T0 has two replicates (A-B) and the other treatments have three replicates (A-C). Brightness of bands indicates how abundant a taxon was within its community. Red numbers represent bands that were sequenced. (DOCX) [file pone.0215872.s004.docx]

Nanosize fraction (2-10µm)


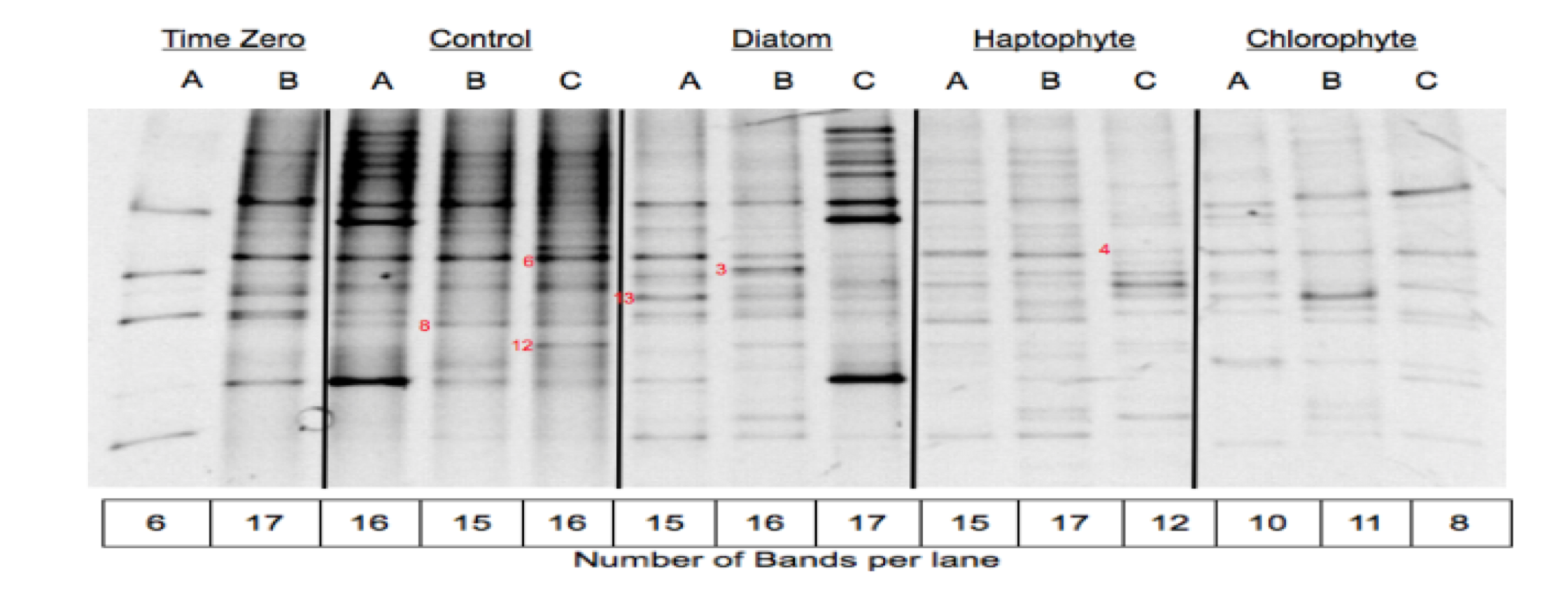

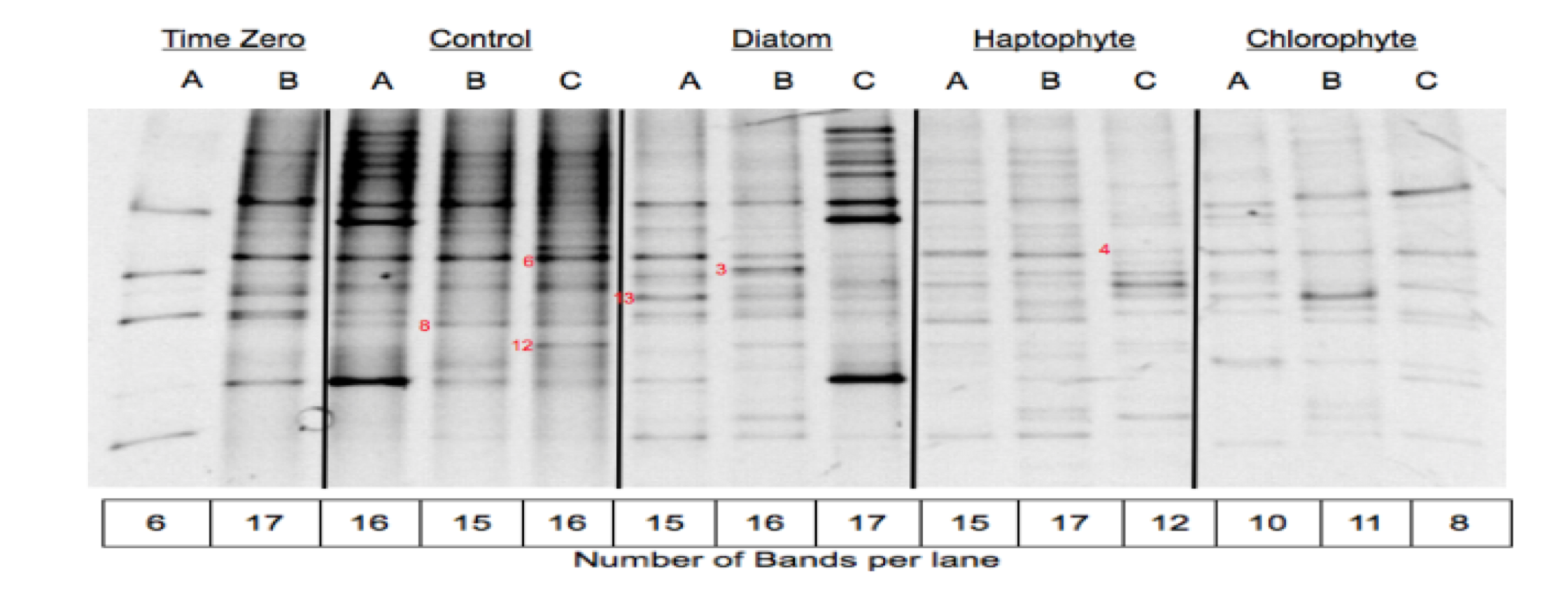


Microsize fraction (10-80µm)


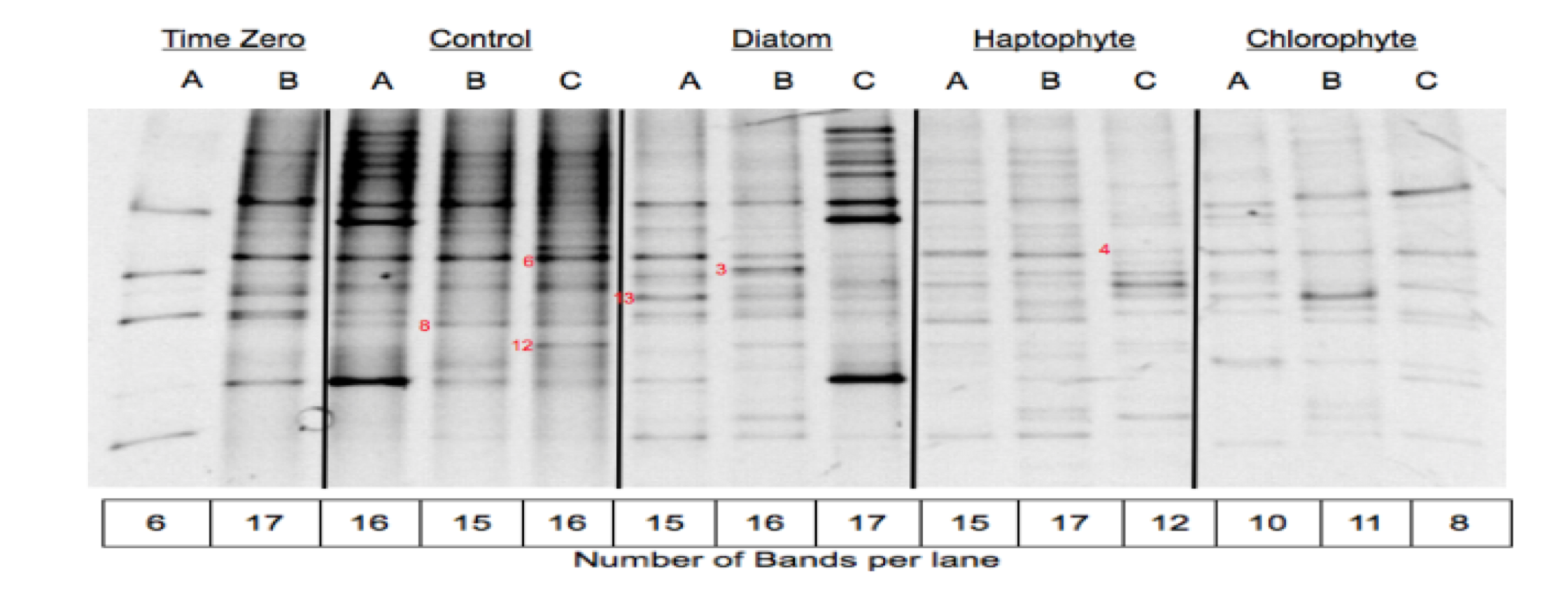

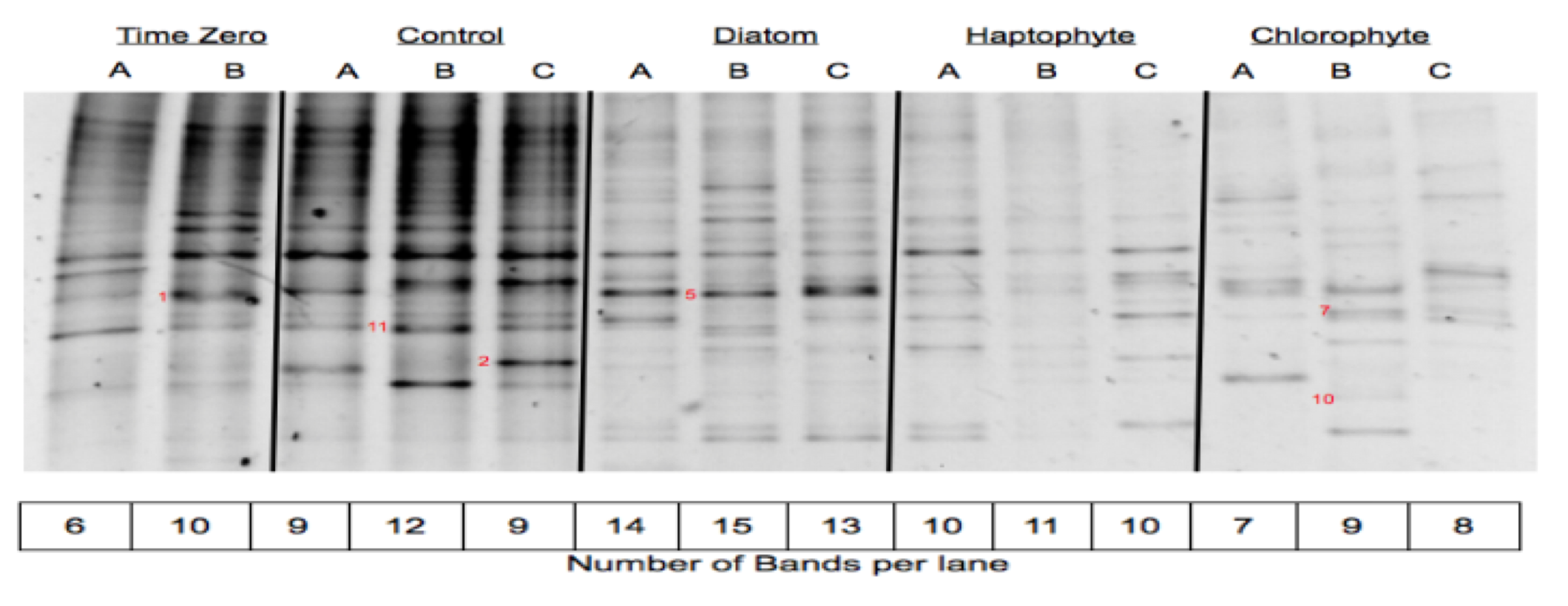


**S4 Fig. DGGE of bottom-up experiment 1 (BU 1) using Spirotrichea primers shows high variability among replicates.** Each lane presents a replicate of T0, control, and three bloom treatments. T0 has two replicates (A-B) and the other treatments have three replicates (A-C). Brightness of bands indicates how abundant a taxon was within its community. Red numbers represent bands that were sequenced.
