# Supplementary material for: Incubation and grazing effects on spirotrich ciliate diversity inferred from molecular analyses of microcosm experiments
Source: PLoS One. 2019 May 6;14(5):e0215872. doi: 10.1371/journal.pone.0215872 (PMC6502329; doi:10.1371/journal.pone.0215872)
Supplement: S5 Fig — Other notes as in S4 Fig. (DOCX) [file pone.0215872.s005.docx]

Nanosize fraction (2-10µm)


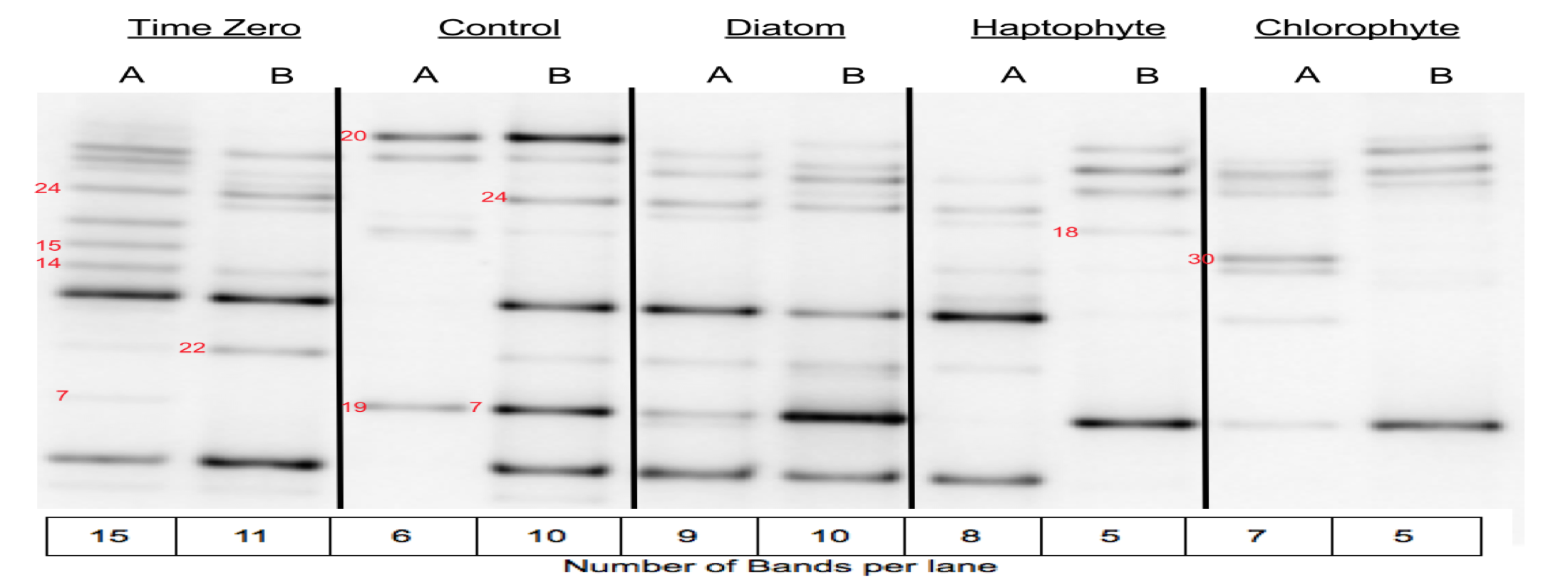


Microsize fraction (10-80µm)


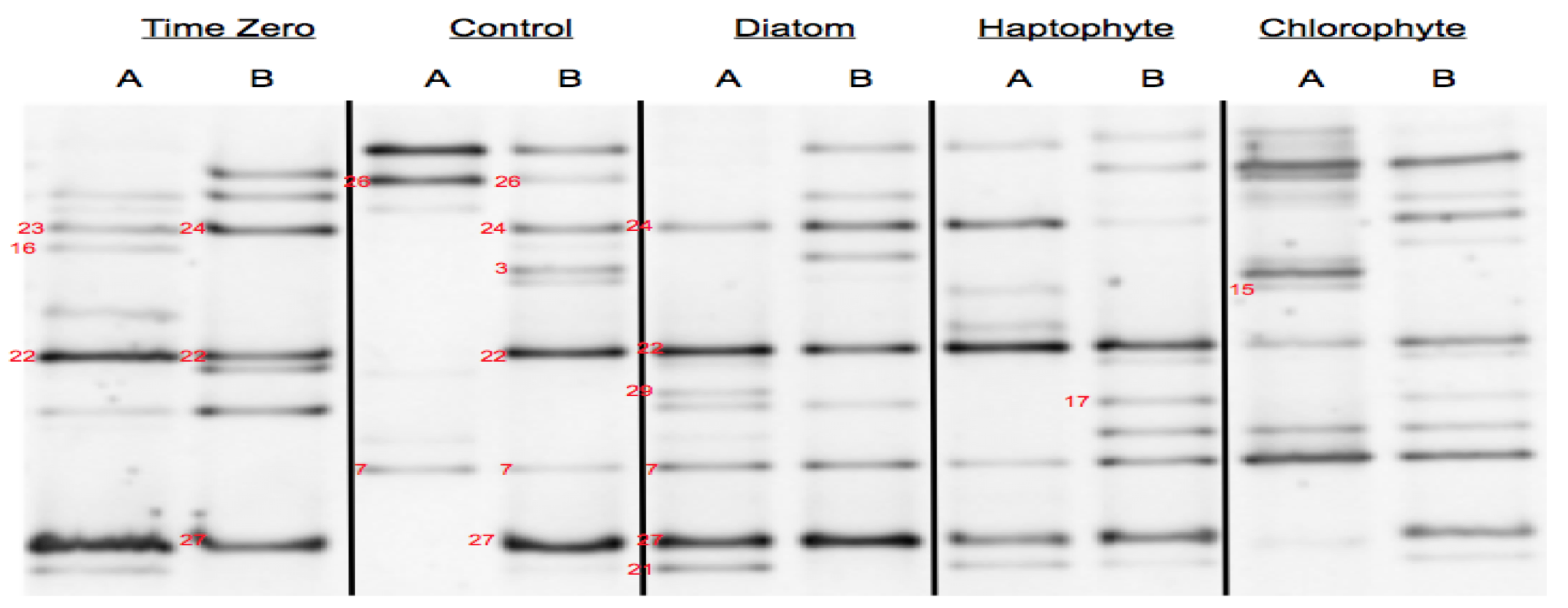


**S5 Fig. DGGE of bottom up Experiment 2 (BU 2) using Spirotrichea primers shows high variability among replicates.** Other notes as in Figure S4.
