# Supplementary material for: Incubation and grazing effects on spirotrich ciliate diversity inferred from molecular analyses of microcosm experiments
Source: PLoS One. 2019 May 6;14(5):e0215872. doi: 10.1371/journal.pone.0215872 (PMC6502329; doi:10.1371/journal.pone.0215872)
Supplement: S7 Fig — The reference morphospecies are in red for Tintinnida, in green for Choreotrichida and in blue for Oligotrichia. In grey are previously sequenced DGGE haplotype [54,55,70,72] and in black are outgroup morphospecies. Sequences were aligned with Muscle and tree was built using the GRTGAMMAI parameter in RaxML. (DOCX) [file pone.0215872.s007.docx]

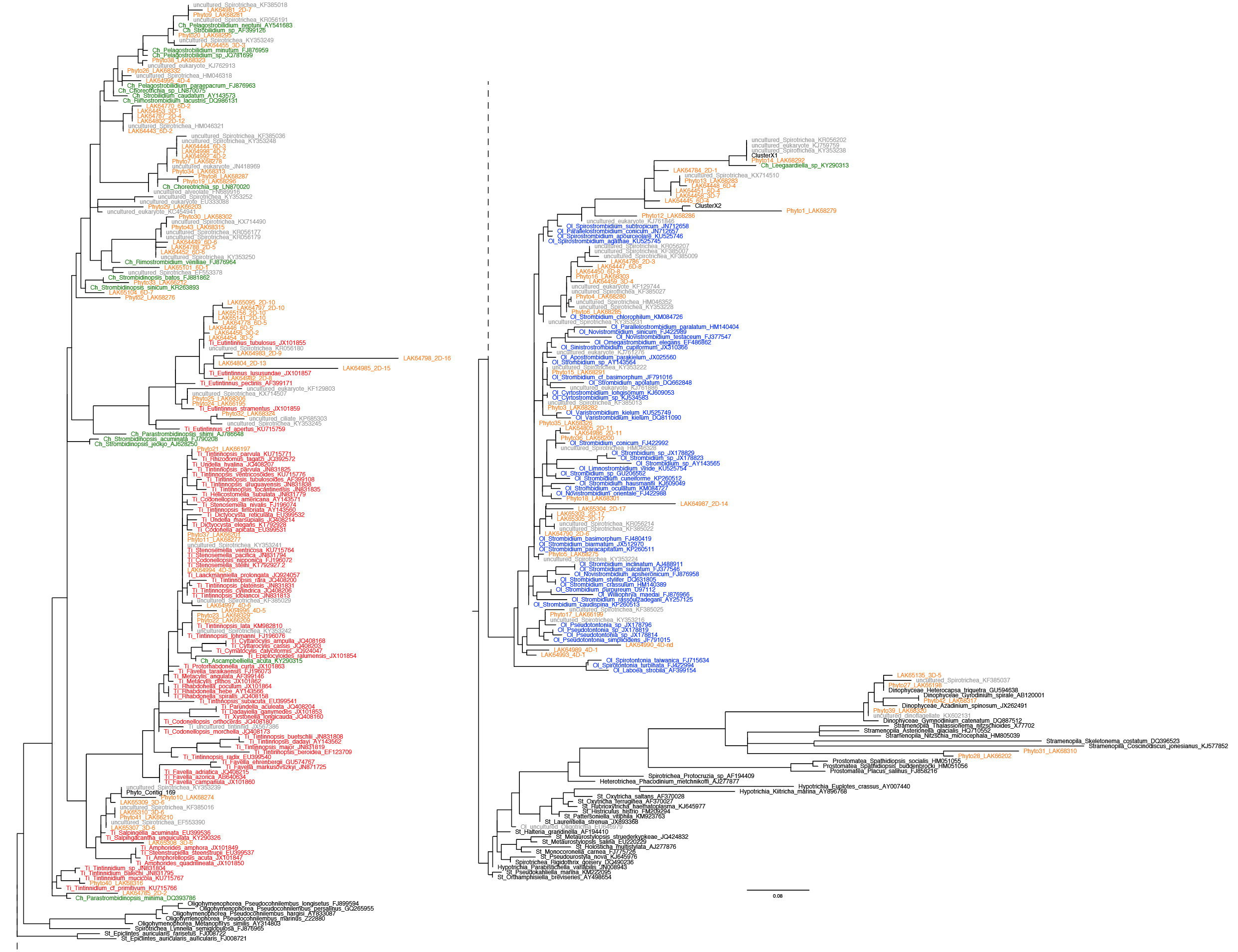


**S7 Fig. Phylogeny of the DGGE haplotypes (orange) reveals diversity of lineages generated using spirotrich ciliate DGGE primers.** The reference morphospecies are in red for Tintinnida, in green for Choreotrichida and in blue for Oligotrichia. In grey are previously sequenced DGGE haplotype [54,55,70,72] and in black are outgroup morphospecies. Sequences were aligned with Muscle and tree was built using the GRTGAMMAI parameter in RaxML
