# Supplementary material for: Incubation and grazing effects on spirotrich ciliate diversity inferred from molecular analyses of microcosm experiments
Source: PLoS One. 2019 May 6;14(5):e0215872. doi: 10.1371/journal.pone.0215872 (PMC6502329; doi:10.1371/journal.pone.0215872)
Supplement: S8 Fig — Distribution of dominant (A) stramenopiles in our phytoplankton microcosm experiment analyzed using high throughput sequencing and SAR-specific primers. The left part of the graph shows each replicate for each size fraction and for each incubation condition, while the right part represents only the incubations conditions (replicates and size have been pooled together). The * denote samples with less than 50,000 rarefied reads. (DOCX) [file pone.0215872.s008.docx]

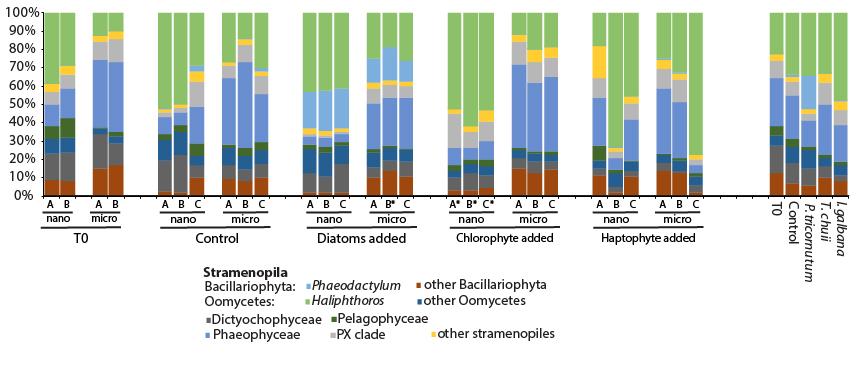


**S8 Fig. Distribution of dominant stramenopiles in our phytoplankton microcosm experiment analyzed using high throughput sequencing and SAR-specific primers.** The left part of the graph shows each replicate for each size fraction and for each incubation condition, while the right part represents only the incubations conditions (replicates and size have been pooled together). The * denote samples with less than 50,000 rarefied reads**.**
