# Supplementary material for: Incubation and grazing effects on spirotrich ciliate diversity inferred from molecular analyses of microcosm experiments
Source: PLoS One. 2019 May 6;14(5):e0215872. doi: 10.1371/journal.pone.0215872 (PMC6502329; doi:10.1371/journal.pone.0215872)
Supplement: S1 Table — (DOCX) [file pone.0215872.s009.docx]

**S1 Table.** Primer sets used in this study.

| **Clade** | **Primer** | **Sequence** | **Amplicon length (bp)** | **reference** |
| --- | --- | --- | --- | --- |
| Spirotrich Ciliates | 152+ | TTACATGGATAACCGTGGTA | ~350 | Doherty et al. 2007, Tamura et al. 2011 |
|  | 528- | CCCGGCCCGTTATTTCTTGT |  |  |
| ‘All’ eukaryotes | 960F | GGCTTAATTTGACTCAACRCG | ~300 | Gast et al 2004 |
|  | 1200R | GGGCATCACAGACCTG |  |  |
| SAR clade | SAR_F | AYTCAGGGAGGTAGTGACAAG | ~150 | This study, Sisson et al 2018 |
|  | SAR_R | RACTACGAGCTTTTTAACTGC |  |  |
| For DGGE | GC clamp | CGCCCGCCGCGCCCCGCGCCCGTCCCGCCGCCCCCGCCC | ~400 | Tamura et al. 2011 |
| For HTS | Forward hook | TCGTCGGCAGCGTCAGATGTGTATAAGAGACAG | ~220 | URI |
|  | Reverse hook | GTCTCGTGGGCTCGGAGATGTGTATAAGAGACAG |  |  |
