# Supplementary material for: Incubation and grazing effects on spirotrich ciliate diversity inferred from molecular analyses of microcosm experiments
Source: PLoS One. 2019 May 6;14(5):e0215872. doi: 10.1371/journal.pone.0215872 (PMC6502329; doi:10.1371/journal.pone.0215872)
Supplement: S2 Table — (DOCX) [file pone.0215872.s010.docx]

**S2 Table.** Number of abundant spirotrich (top) and eukaryotic (bottom) species (bands) in each replicate during the top down experiments.

| Spiro | T0 | Control | | | | | | ‘Natural’ Predation | | | | | | ‘High’ predation | | | | | |  |
| --- | --- | --- | --- | --- | --- | --- | --- | --- | --- | --- | --- | --- | --- | --- | --- | --- | --- | --- | --- | --- |
|  |  | a | b | c | **tot** | **All** | **M** | a | b | c | **tot** | **All** | **M** | a | b | c | **tot** | **All** | **M** |  |
| TD1 | **25** | 14 | 17 | 16 | **19** | 12 | 15.7 | 3 | 6 | 13 | **13** | 3 | 7.3 | 12 | 11 | 10 | **15** | 5 | 11 |  |
| TD2 | **8** | 5 | 1 | 4 | **5** | 1 | 3.3 | 4 | 5 | 6 | **6** | 3 | 5 | 1 | - | - | **1** | - | - |  |
| TD3 | **10** | 5 | 2 | 2 | **6** | 1 | 3 | 7 | 5 | 2 | **9** | 1 | 4.7 | 3 | 6 | - | **6** | - | 4.5 |  |
| Euk | **T0** | **Control** | | | | | | **‘Natural’ predation** | | | | | | **‘High’ predation** | | | | | | |
|  |  | a | b | c | **tot** | **All** | **M** | a | b | c | **tot** | **All** | **M** | a | b | c | **tot** | **All** | **M** | |
| TD1 | **10** | 4 | 10 | 13 | **16** | 4 | 9 | 3 | 11 | 9 | **12** | 3 | 7.7 | 9 | 8 | 9 | **10** | 8 | 8.7 | |
| TD2 | **12** | 11 | 14 | 10 | **15** | 9 | 11.7 | 9 | 9 | 9 | **9** | 9 | 9 | 8 | 8 | - | **9** | - | 5.3 | |
| TD3 | **25** | 16 | 7 | 9 | **20** | 4 | 10.7 | 23 | 13 | 13 | **27** | 9 | 16.3 | 16 | 12 | 14 | **21** | 9 | 14 | |

a, b, and c represent the replicates, ‘tot’ the total species richness observed for the treatment, ‘All’ the number of species shared across the three replicates and, ‘M’ the average of bands across replicates.
