# Supplementary material for: Incubation and grazing effects on spirotrich ciliate diversity inferred from molecular analyses of microcosm experiments
Source: PLoS One. 2019 May 6;14(5):e0215872. doi: 10.1371/journal.pone.0215872 (PMC6502329; doi:10.1371/journal.pone.0215872)
Supplement: S3 Table — (DOCX) [file pone.0215872.s011.docx]

**S3 Table.** Number of abundant nanosize (top) and microsize (bottom) spirotrich ciliate species (band) during the bottom-up experiments.

| nano | T0 | | | | Control | | | | | Diatoms | | | | | Haptophyte | | | | | Chlorophyte | | | | |
| --- | --- | --- | --- | --- | --- | --- | --- | --- | --- | --- | --- | --- | --- | --- | --- | --- | --- | --- | --- | --- | --- | --- | --- | --- |
|  | A | B | **S** | **M** | A | B | C | **S** | **M** | A | B | C | **S** | **M** | A | B | C | **S** | **M** | A | B | C | **S** | **M** |
| BU1 | 6 | 12 | **4** | 9 | 13 | 13 | 15 | **14** | 13.7 | 13 | 16 | 13 | **13** | 14 | 13 | 14 | 13 | **10** | 13.3 | 10 | 11 | 10 | **8** | 10.3 |
| BU2 | 12 | 10 | **8** | 11 | 6 | 9 | - | **4** | 7.5 | 9 | 9 | - | **6** | 9 | 8 | 7 | - | **3** | 7.5 | 8 | 5 | - | **2** | 6.5 |
| BU3 | 13 | 5 | **4** | 9 | 7 | 8 | 1 | **1** | 5.3 | 2 | 5 | 7 | **3** | 4.7 | 3 | 6 | 7 | **3** | 5.3 | 8 | 6 | 7 | **6** | 7 |
| micro | **T0** | | | | **Control** | | | | | **Diatoms** | | | | | **Haptophyte** | | | | | **Chlorophyte** | | | | |
|  | A | B | **S** | **M** | A | B | C | **S** | **M** | A | B | C | **S** | **M** | A | B | C | **S** | **M** | A | B | C | **S** | **M** |
| BU1 | 11 | 16 | **8** | 13.5 | 9 | 11 | 13 | **10** | 11 | 13 | 14 | 13 | **13** | 13.3 | 12 | 10 | 13 | **11** | 11.7 | 8 | 9 | 7 | **7** | 8 |
| BU2 | 10 | 7 | **5** | 8.5 | 6 | 10 | - | **3** | 8 | 9 | 9 | - | **5** | 9 | 8 | 10 | - | **5** | 9 | 12 | 12 | - | **4** | 12 |
| BU3 | 8 | 1 | **1** | 4.5 | 7 | 5 | 7 | **5** | 6.3 | 1 | 8 | 3 | **3** | 4 | 6 | 7 | 6 | **4** | 6.3 | 9 | 7 | 5 | **5** | 7 |

A, B, C represent the replicates, M the average of species, and S the number of species shared by at least two of the replicates given the high variability observed in these experiments.
