# Supplementary figures and images for: Incubation and grazing effects on spirotrich ciliate diversity inferred from molecular analyses of microcosm experiments
Source: PLoS One. 2019 May 6;14(5):e0215872. doi: 10.1371/journal.pone.0215872 (PMC6502329; doi:10.1371/journal.pone.0215872)

**S4 Table.** Top Blast hits for the copepod and phytoplankton microcosm experiments.


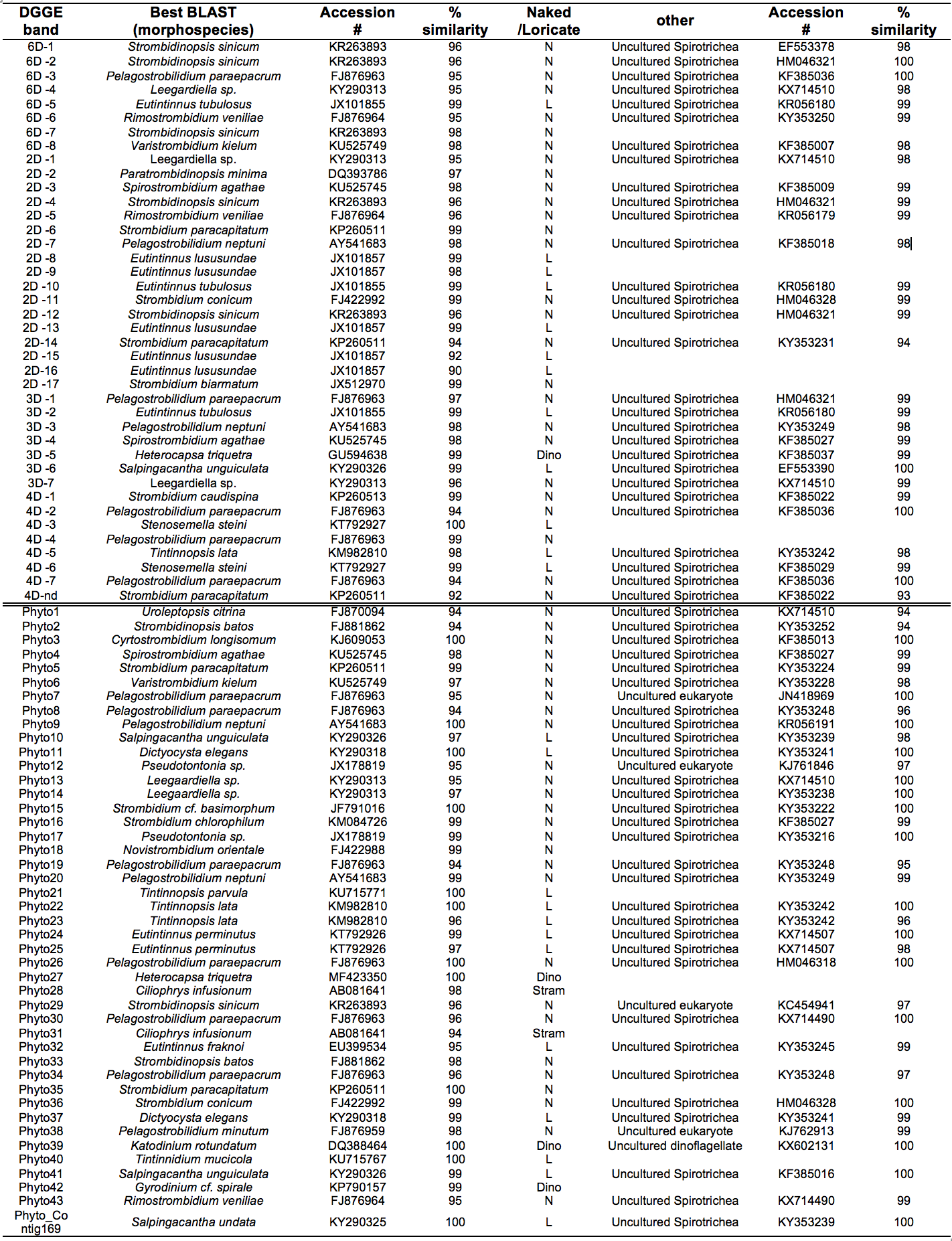

Supplement: S4 Table — (DOCX) [file pone.0215872.s012.docx]
