# Supplementary material for: Incubation and grazing effects on spirotrich ciliate diversity inferred from molecular analyses of microcosm experiments
Source: PLoS One. 2019 May 6;14(5):e0215872. doi: 10.1371/journal.pone.0215872 (PMC6502329; doi:10.1371/journal.pone.0215872)
Supplement: S5 Table — (DOCX) [file pone.0215872.s013.docx]

**S5 Table.** Copepods abundance during the copepod experiments.

| **Microcosm** | **Treatment** | **Copepods in bag (start)** | **Copepods in bag (end)** |  | |
| --- | --- | --- | --- | --- | --- |
| TD 1 | C 1 | 0 | Nd |  |  |
| TD 1 | C 2 | 0 | Nd |  |  |
| TD 1 | C 3 | 0 | Nd |  |  |
| TD 1 | N 1 | 5 | Nd |  |  |
| TD 1 | N 2 | 5 | Nd |  |  |
| TD 1 | N 3 | 5 | Nd |  |  |
| TD 1 | H 1 | 10 | Nd |  |  |
| TD 1 | H 2 | 10 | Nd |  |  |
| TD 1 | H 3 | 10 | Nd |  |  |
| TD 2 | C 1 | 0 | 2 |  |  |
| TD 2 | C 2 | 0 | 11 | 7.3 |  |
| TD 2 | C 3 | 0 | 9 |  |  |
| TD 2 | N 1 | 5 | 13 |  |  |
| TD 2 | N 2 | 5 | 26 | 21.3 |  |
| TD 2 | N 3 | 5 | 25 |  |  |
| TD 2 | H 1 | 10 | 17 |  |  |
| TD 2 | H 2 | 10 | 10 | 22 |  |
| TD 2 | H 3 | 10 | 39 |  |  |
| TD 3 | C 1 | 0 | 16 |  | |
| TD 3 | C 2 | 0 | 27 | 18.3 | |
| TD 3 | C 3 | 0 | 12 |  | |
| TD 3 | N 1 | 5 | 8 |  | |
| TD 3 | N 2 | 5 | 21 | 12 | |
| TD 3 | N 3 | 5 | 7 |  | |
| TD 3 | H 1 | 10 | 9 |  | |
| TD 3 | H 2 | 10 | 12 | 9.7 | |
| TD 3 | H 3 | 10 | 8 |  | |
