# Supplementary material for: Incubation and grazing effects on spirotrich ciliate diversity inferred from molecular analyses of microcosm experiments
Source: PLoS One. 2019 May 6;14(5):e0215872. doi: 10.1371/journal.pone.0215872 (PMC6502329; doi:10.1371/journal.pone.0215872)
Supplement: S6 Table — (DOCX) [file pone.0215872.s014.docx]

**S6 Table.** PERMANOVA results (R^2^) from HTS SAR and Spirotrichea data with 999 permutations show (1) community composition related to size and treatment. None post hoc with Bonferroni correction are significant for each pair comparison

| **index** | **Size** | **Size** | **Treatment** | **Size:Treatment** |
| --- | --- | --- | --- | --- |
| **SAR** |  |  |  |  |
| **Bray** | Both | **0.32***** | **0.30***** | ns |
|  | Nano | na | **0.59***** | na |
|  | Micro | na | **0.55***** | na |
| **Unifrac** | Both | **0.35***** | **0.28***** | ns |
|  | Nano | na | **0.51*** | na |
|  | Micro | na | **0.56**** | na |
| **Spirotrichea** |  |  |  |  |
| **Bray** | Both | **0.20***** | **0.32***** | ns |
|  | Nano | na | **0.55**** | na |
|  | Micro | na | **0.48**** | na |
| **Unifrac** | Both | **0.26**** | **0.36***** | ns |
|  | Nano | na | **0.48*** | na |
|  | Micro | na | **0.49**** | na |

Note: Nano: 2-10µm and Micro 10-80µm; *** P<0.001 ** P<0.01 * P<0.05; ns = not significant; na = not applicable
